# Genome-wide mining and characterization of MATE transporters in Coriandrum sativum L.

**DOI:** 10.22099/mbrc.2024.49840.1954

**Published:** 2024

**Authors:** Deepu Mathew, Ravisankar Valsalan, M. Shijili

**Affiliations:** Bioinformatics Centre, Kerala Agricultural University, Thrissur-680 656, India

**Keywords:** Coriander, Genome annotation, Homeostasis, Multidrug and Toxic Compound Extrusion, Transport protein

## Abstract

Multidrug and Toxic Compound Extrusion (MATE) proteins are responsible for the transport of a wide range of metabolites out of plant cells. This helps to protect the cells from toxins and other harmful compounds. MATE proteins also play a role in plant development, by regulating the transport of hormones and other signalling molecules. They transport a wide variety of substances, including organic acids, plant hormones, flavonoids, alkaloids, terpenes and other secondary metabolites. MATE proteins are thought to play similar roles in Coriander, in addition to stress responses. The MATE genes in the coriander genome have been identified and characterized. Detailed genome homology search and domain identification analysis have identified 91 MATE proteins in the genome assembly of coriander. A phylogenetic analysis of the identified proteins divided them into five major clades. The functions of the transporters in each cluster were predicted based on the clustering pattern of the functionally characterized proteins. The amino acid sequences, exon-intron structures and motif details of all the 91 proteins are identified and described. This is the first work on the MATE transporters in coriander and the results deliver clues for the molecular mechanisms behind the stress responses and secondary metabolite transport in coriander.

## INTRODUCTION

Coriander (*Coriandrum sativum*, Apiaceae, 2n=2x=22), also known as Chinese parsley, is a globally important herbal spice cum vegetable crop. Global coriander production tripled (http://faostat3.fao.org/) between 1994 and 2020, with Asia accounting for 71.4% of the total. This crop is known by two names: cilantro for its leaves and stems, and coriander for its dried seeds. All parts of the plant are edible and have different flavors. The leaves and stems have a fresh, citrusy flavor, while the seeds have a warm, nutty flavor [1]. 

Abiotic stresses and the contaminants in soil, water, and air, have adverse influence on both physiological and morphological traits in coriander [2, 3]. Tolerance to these factors is a product of the integration of multiple biological processes, from the genetic makeup of the organism to the biochemistry of individual cells. Although it is obvious that coriander plant genetics plays an important role in the response mechanisms to these stresses, little is known on the multifaceted roles of various genes.

MATE transporters are a type of secondary active transport proteins found in all the three domains of life: archaea, bacteria, and eukaryotes. These transporters are involved in the export of a wide range of drugs, toxins, and other harmful substances from the cells, utilizing the electrostatic potential difference generated by Na^+^/H^+^ ions across the membrane [4]. Studies following the discovery of NorM, the first MATE protein, from *Vibrio parahaemolyticus* in 1998, have shown that MATE proteins from other organisms have a sequence similarity of approximately 40% [5-7]. NorM protein has 12 transmembrane domains that are arranged in two bundles of 6 helices each (TM1-TM6 and TM7-TM12). The helices are arranged in two layers along the lipid membrane, forming a large cavity that is open to the extracellular space [8,9,10]. Most of the MATE transporters are relatively smaller, accommodating 400 to 700 amino acids. Unlike other multidrug transporter families, they lack signature sequences, which makes their identification difficult [11, 12]. The first mammalian MATE proteins were found in humans and mice. The genes for *SLC47A1* and *SLC47A2*, which encode these proteins, are expressed in the kidney and liver [13,14]. Since the first report of a MATE protein, research on MATE members has expanded to a wide range of living organisms. 

Plants have a more diverse set of MATE proteins than bacteria or animals [7]. These transporters are found in all the plant cells and are involved in a wide array of important functions, including the transport of secondary metabolites, detoxification of harmful substances, resistance to disease, and tolerance to heavy metals [7, 15]. So far, *Arabidopsis* and rice are found to possess 56 and 52 MATE proteins, respectively [11, 16]. The detailed structure and function of MATE proteins have been reported in a number of plant species, including pepper (*Capsicum annuum*), apple [17], chickpea [18], potato [19], tomato [20], wheat [21], corn [22], tobacco [23], and mangrove plants [24]. The highest number of MATE proteins reported so far from a plant species is from the allohexaplooid *Triticum aestivum*, where a total of 211 sequences were discovered.

In addition to the above-mentioned functions, their vital roles in ABA efflux, iron translocation, and aluminum detoxifcation are well established [25-28]. In *Arabidopsis*, the MATE protein AtDTX41/TT12, which is a flavonoid transporter, helps export two important flavonoids: glycosylated flavan-3-ol monomer and epicatechin 3́-O-glucoside [29-31]. Likewise, in *Arabidopsis*, it has been shown that overexpressing the cotton *DTX*/*MATE* gene causes and increase in the tolerance of plants to salt, drought and cold [32]. The HvAACT1 MATE protein in barley is located on the plasma membrane of epidermal cells in the roots. It is responsible for the secretion of citrate in response to aluminum, which is a key part of how plants detoxify aluminum [33].

 MATE transporters in coriander plants are thought to play dynamic roles in the transport of a wide variety of substances including secondary metabolites, and molecular adaptations to various stresses and contaminants. The *MATE* genes in coriander are yet to be identified and functionally characterized. The mining of *MATE* proteins from coriander genome followed by comprehensive analyses on evolutionary relationships, gene structure and motifs, were performed. Based on these results, we discuss the adaptive strategies by the plant to cope up with the stresses.

## MATERIALS AND METHODS

**Genome assembly selection and annotation: **Genome assembly of *C.sativum *was retrieved from Genome Sequence Archive in BIG Data Center, Beijing Institute of Genomics (BIG), Chinese Academy of Sciences (Acc. no. CRA001654). The genome assembly was used to create a custom repeat library by identifying and modeling repetitive elements. RepeatModeler, a software package for constructing repeat libraries, used three de novo repeat finding programs, namely, RECON, RepeatScout, and LtrHarvest/Ltr_retriever to identify and model repetitive elements in the genome. The custom repeat library developed for the genome was used in RepeatMasker (https://www.repeatmasker.org/) and the repeat elements were masked. This was followed by gene prediction using Augustus 3.4.0 tool [34] on the repeat masked genomes, where *Arabidopsis thaliana *was used as the training set.

**Identification of** **MATE** **sequences: **Twenty-five amino acid sequences of MATE proteins from eight plant species (*Arabidopsis thaliana*, *Glycine max, Nicotiana tabacum*, *Oryza sativa*, *Vitis vinifera*, *Malus domestica*, *Medicago truncatula*, *Eucalyptus camaldulensis* and *Brassica oleracea*) were selected from the UniProt/GenPept database (Table S1) [35, 36]. The hidden Markov model (HMM) profile (ID: PF01554) of the MATE gene family was downloaded from the Pfam database. A query consisting of the manually curated set of sequences and the downloaded Pfam profile was used in HMMER [37]. HMMSEARCH program was used to search for protein sequences homologous to MATE proteins. The protein sequences were predicted using Augustus tool, and the output from HMMSEARCH were manually confirmed using BLAST. Resulted sequences were then used to generate a preliminary dataset. The CD-HIT tool [38] was then used to remove redundant sequences with an identity of at least 90%. To improve the accuracy of gene finding, putative MATE protein sequences were manually filtered as described. The presence of the conserved domains were detected using Conserved Domain Database (CDD) at NCBI [39] and Simple Modular Architecture Research Tool (SMART) [40]. The presence of transmembrane helices in the proteins was predicted using TMHHM tool [41]. The obtained sequences were analyzed to determine whether they contained MATE domains and transmembrane domains, which are characteristic features of coriander MATE proteins. The sequences were thus manually selected and the final dataset was prepared. 

**Phylogenetic analysis: **To perform the phylogenetic analysis, sequences identified from the genome was used in conjunction with 25 sequences of previously described MATE genes from various genomes. MAFFT v.7 [42] with default parameters was used for the multiple sequence alignment. The results from the alignment were employed in ProtTest 3.4.2 [43] to detect the top model for phylogenetic tree construction. The top model, JTT+G+F, was used to build the phylogenetic tree using 1000 bootstrap replications in RAxML 8.2.12 [44].

**Exon-intron structure and motif elucidation: **Gene structure (Exon-Intron) analysis was performed via the Gene Structure Display Server (GSDS) [45] with default setting. Motifs were identified from the selected MATE proteins using MEME suite [46] with settings described by Shijili et al. [24]. 

**Subcellular localization prediction: **The protein sequences obtained from the study were subjected to subcellular localization prediction using the server version of DeepLoc-2.0 [47]. High-quality model was chosen for the analysis and the long output format was selected.

## RESULTS

A repeat element library for *Coriandrum sativum* was created using RepeatModeler, and the identified repeat elements were masked using RepeatMasker. The repeat sequences identified was 81.16%. Augustus, the ab-initio gene prediction tool, predicted 42,462 protein-coding genes in the repeat-masked genome of *C. sativum*.

An exhaustive search of the coriander genome using HMMER identified 91 MATE proteins. Proteins identified in this study were named using the short form of their scientific names, followed by "MATE" and the sequence number, such as CsMATE1, CsMATE2 and so on. The proteins were highly diverse in length, with amino acid sequences ranging from 91 to 1194 residues (File S1).

**Figure 1:**
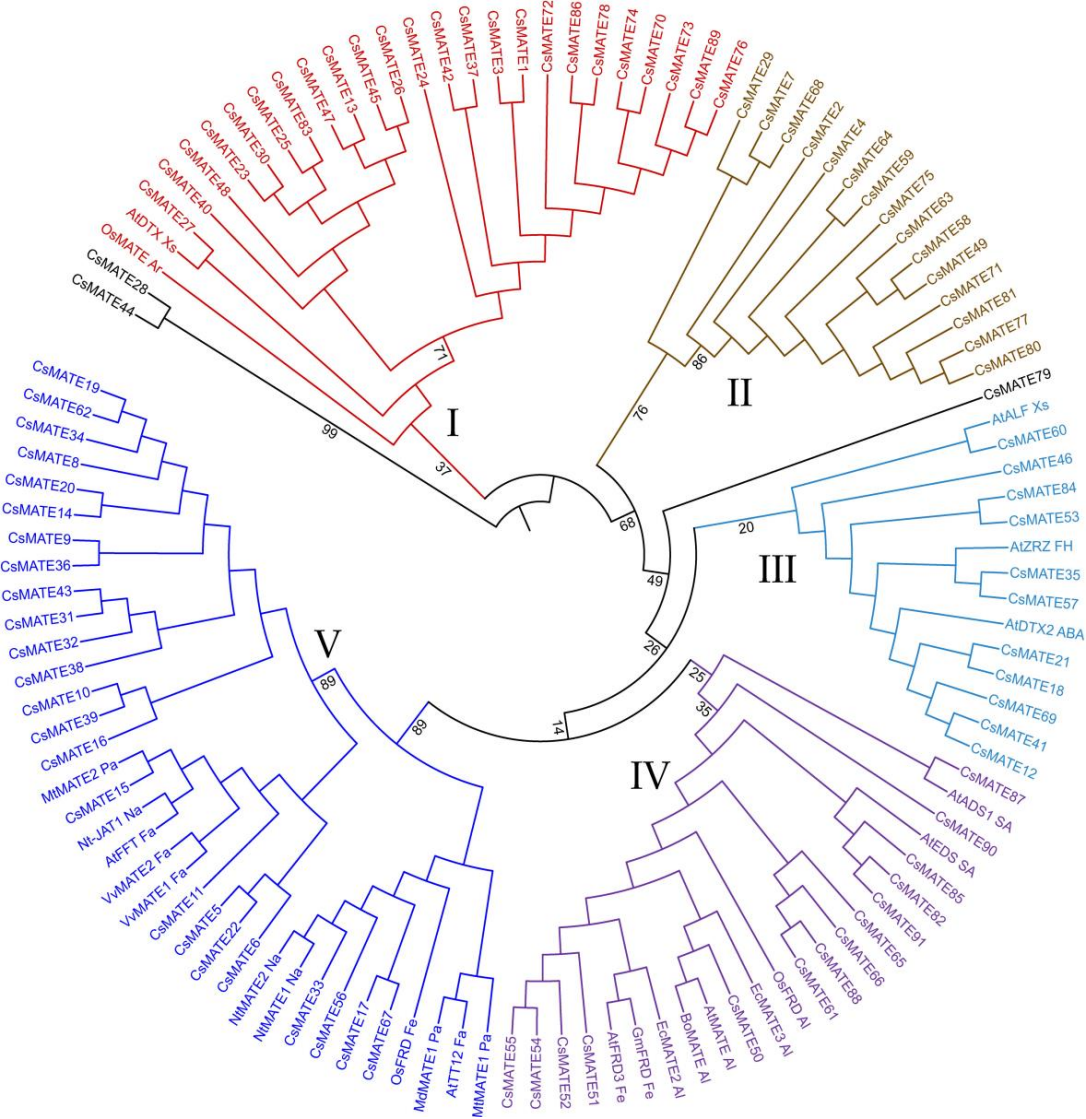
Phylogenetic tree of the MATE transporters identified from coriander. The major clusters are color-coded and numbered, and bootstrap values (%) are shown along the major branches.

Full length sequences of 91 proteins identified from this study, along with that of 25 previously functionally described MATE proteins from other plants were used for the phylogenetic analysis. The phylogenetic tree was divided into five major clusters, labeled I-V. The clusters contained 26, 15, 14, 23 and 35 proteins, respectively (Fig. 1). The clusters had medium to high bootstrap support. The new proteins were hypothesized to have similar functions to the previously described MATE transporters in each cluster. Accordingly, the proteins in cluster I had proteins contributing in Arsenic efflux and Xenobiotic efflux. Surprisingly, Cluster II had none of the previously described proteins. Cluster III was accommodating proteins, which play roles in ABA signaling and iron homeostasis while cluster IV had proteins found in salicylic acid signaling, aluminum detoxification and iron translocation. Cluster V was the biggest, accommodating the proteins having roles in the accumulation of nicotine, flavonoids and proanthocyanidins. 

**Figure 2:**
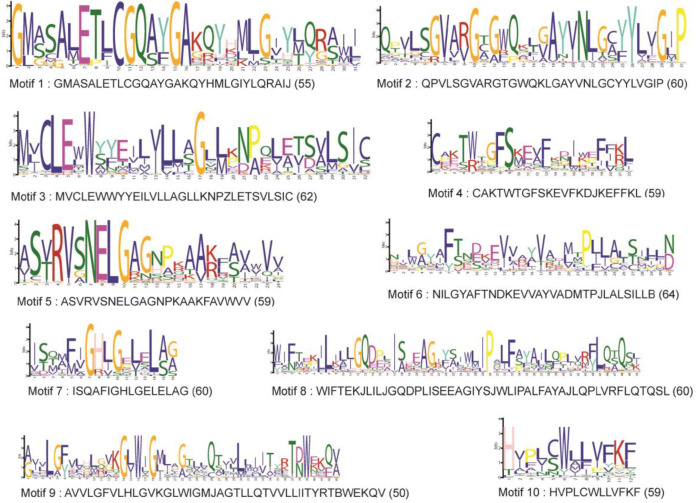
The ten major motifs identified in coriander MATE proteins are described. The motif number and consensus sequence are provided, along with the number of occurrences of each motif in parenthesis.

Exon-intron analysis was performed to further understand the structural and functional characteristics of MATE genes in coriander. The genes were found to have one to fourteen exons, except in *CsMATE1* and *CsMATE55*, which had 22 and 19 exons, respectively. Among the proteins identified, six were mono-exonic. Most of the proteins had seven, eight or nine exons whose numbers were 13, 18 and 10, respectively. The exon-intron structures of all the 91 proteins identified in this study are represented in Figure S1. MEME analysis of coriander MATE proteins revealed that they have ten major motifs, all of which were present in more than 50 sequences (Fig. 2). Subcellular localization of the identified proteins were also analysed. From the results it was found that 50 out of 91 proteins were localised in cell membrane, 22 were localised to Lysosome/Vacuole and the remaining proteins were localised to endoplasmic reticulum.

## DISCUSSION

The MATE family is a large group of transport proteins that are essential for many physiological processes in plants. Recent genome wide studies have identified MATE proteins in a variety of plant species, where they play diverse roles in stress responses, plant growth, and transmembrane transport. These proteins play a crucial role in transporting substances across cellular membranes, contributing to stress tolerance, regulating plant development, and maintaining cellular balance. A better understanding of how MATE genes function and are regulated could help us develop plant lines with improved yield and stress tolerance [17,19, 20-22,48-50]. This is the first attempt to detect and functionally characterize the MATE proteins in coriander, an economically important herbaceous spice crop.

From the genome assembly of *Coriandrum sativum *[1], this study has identified 91 novel MATE genes. The results of the phylogenetic, exon-intron, and motif pattern analyses have revealed new information about the evolutionary history, functional properties, and structural features of the MATE proteins in coriander. 

The length of the MATE proteins ranged from 91 to 1194 amino acids, which was similar to the lengths reported in other plant species such as soybean [51], apple [17], *Arabidopsis* [11], rice [52], which were found harboring 80-593, 406-712, 400-700 and 469-575 amino acids, respectively. The number of MATE proteins identified in coriander is higher than the numbers reported from *Arabidopsis*, potato, maize, capsicum, rice and tomato, which have 56 proteins from ~135 Mb genome [16], 48 proteins from ~674 Mb genome [53], 49 proteins from ~2.3 Gb genome [22], 42 proteins from ~3.5 Gb genome [19], 46 proteins from ~380 Mb genome [48] and 67 proteins from ~950 Mb genome [20], respectively. The number of MATE proteins in coriander was lesser than the numbers reported from soybean, *Nicotiana tabacum *and wheat comprising of 117 proteins from ~978 Mb genome [54], 138 proteins from ~4.5 Gb genome [23] and 211 proteins from ~16 Gb genome [21], respectively. Although the number of MATE proteins in a genome is not correlated with genome size, polyploidy is known to increase their abundance. The coriander genome has a greater number of MATE proteins than genomes of similar size, such as maize. This disparity suggests that coriander has a higher gene density and that MATE genes may play diverse roles in this crop. 

The classification MATE proteins into five major clades through phylogenetic analysis was similar to the findings of few former works [20, 24, 51]. The first cluster had 26 proteins, containing two previously reported ones. One protein from rice present in this cluster had role in arsenic efflux, and the second protein was from *Arabidopsis*, involved in xenobiotics efflux [11, 55-59]. Because the analysis relies on amino acid sequences, which determine the protein's structure and function, it is hypothesized that the 26 coriander proteins in this cluster are involved in similar functions. Notably, cluster II did not contain any protein sequences identified in previous studies, suggesting their functional dissimilarity to the reference sequences. Hence, these proteins are thought to be involved in dissimilar functions. Cluster III had reference proteins from *Arabidopsis*, which were involved in ABA signaling and iron homeostasis [28, 60-62]. Therefore, it is proposed that the coriander homologs in this cluster play essential roles in similar functions. Clusters IV and V accommodated many reference proteins that were distributed throughout the groups. Cluster IV had nine previously reported proteins, from *Arabidopsis*, *Oryza*, *Brassica oleracea* and *Eucalyptus camaldulensis* involved in salicylic acid signaling, aluminum detoxification and iron translocation [63-67]. Cluster V had 11 reference proteins, from *Arabidopsis*, *Oryza*, *Malus domestica*, *Nicotiana tabacum* and *Medicago truncatula *contributing to the accumulation of nicotine [68], proanthocyanidin and flavonoids and iron translocation [69, 70]. Based on these findings, it is reasonable to propose that these proteins in coriander might serve analogous roles and could be involved in facilitating the transport of secondary metabolites. The higher level of expression of MATE transporters is reported under abiotic stress in rice [48], salt stress in *Arabidopsis*, rice and chickpea [71], heavy metal stress in potato [72], drought, salt and cold stresses in *Arabidopsis* [32] and salt, cadmium and drought stresses in *Gossypium* spp. [73].

Analysis of gene structure has shown that there is a great deal of variation in the exon-intron pattern of genes. Protein motif analysis showed uniform motif pattern, having one to more than ten major motifs. Both exon-intron pattern and motif pattern had no correlation with the function of the protein. The great variability shown in the motif and gene structure analyses among the proteins indicate their functional versatility.

## Conflict of Interest:

The authors declare that there is no conflict of interest regarding the publication of this article.

## Authors’ Contribution:

DM: Conception of idea, experiment planning, data interpretation and manuscript finalization; RV: Execution of experiments and write up of manuscript; SM: Data acquisition and literature search.

## Supplementary materials

Table S1 Details of the previously reported MATE sequences used in this study

Figure S1

File S1
