# Supplementary material for: Genome-wide mining and characterization of MATE transporters in Coriandrum sativum L
Source: Mol Biol Res Commun. 2024;13(3):155–64. doi: 10.22099/mbrc.2024.49840.1954 (PMC11194028; doi:10.22099/mbrc.2024.49840.1954)
Supplement: Table S1 [file mbrc-13-155-s001.pdf]

Table S1: Details of the previously reported MATE sequences used in this study

Sl. No.	Sequence name	Accession id	Species	Sequence length
1	AtALF_Xs	NP_001327615	*Arabidopsis thaliana*	416
2	AtDTX_Xs	Q9SIA5	*Arabidopsis thaliana*	476
3	AtFFT_Fa	F4JTB2	*Arabidopsis thaliana*	514
4	AtTT12_Fa	A0A1I9LM17	*Arabidopsis thaliana*	424
5	AtFRD3_Fe	Q9SFB0	*Arabidopsis thaliana*	526
6	AtMATE_Al	Q9SYD6	*Arabidopsis thaliana*	515
7	AtADS1_SA	A0A1P8AU64	*Arabidopsis thaliana*	227
8	AtDTX2_ABA	Q9FJ87	*Arabidopsis thaliana*	505
9	AtEDS_SA	Q945F0	*Arabidopsis thaliana*	543
10	AtZRZ_FH	Q9SLV0	*Arabidopsis thaliana*	532
11	NtMATE1_Na	A0A1U7XVR3	*Nicotiana tabacum*	500
12	NtMATE2_Na	A3KDM5	*Nicotiana tabacum*	500
13	Nt-JAT1_Na	A0A077LAP2	*Nicotiana tabacum*	507
14	GmFRD_Fe	C1IIX2	*Glycine max*	553
15	OsFRD_Fe	Q75L61	*Oryza sativa*	500
16	OsMATE_Ar	B8AWG0	*Oryza sativa*	500
17	OsFRD_Al	A0A0P0VC55	*Oryza sativa*	599
18	BoMATE_Al	A0A8X7QH85	*Brassica oleracea*	517
19	EcMATE3_Al	BAM68467	*Eucalyptus camaldulensis*	502
20	EcMATE2_Al	BAM68465	*Eucalyptus camaldulensis*	582
21	MdMATE1_Pa	A0A540L9H6	*Malus domestica*	505
22	MtMATE1_Pa	G7KD01	*Medicago truncatula*	504
23	MtMATE2_Pa	F4ZG52	*Medicago truncatula*	501
24	VvMATE1_Fa	F6H6E8	*Vitis vinifera*	489
25	VvMATE2_Fa	E0CUX3	*Vitis vinifera*	493
