# Supplementary material for: Genome-wide mining and characterization of MATE transporters in Coriandrum sativum L
Source: Mol Biol Res Commun. 2024;13(3):155–64. doi: 10.22099/mbrc.2024.49840.1954 (PMC11194028; doi:10.22099/mbrc.2024.49840.1954)
Supplement: Figure S1 [file mbrc-13-155-s002.pdf]

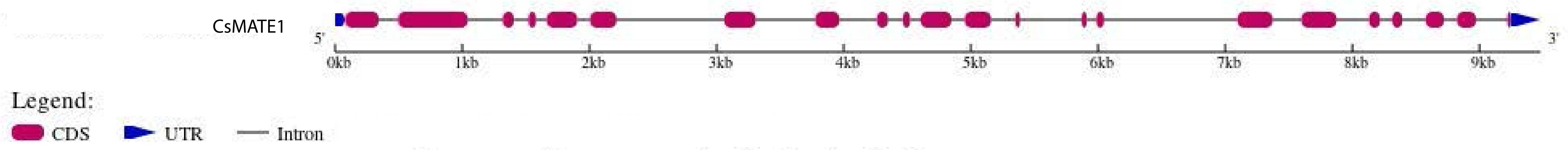
CsMATE1
5'
3'
0kb
1kb
2kb
3kb
4kb
5kb
6kb
7kb
8kb
9kb
Legend:
CDS
UTR
Intron

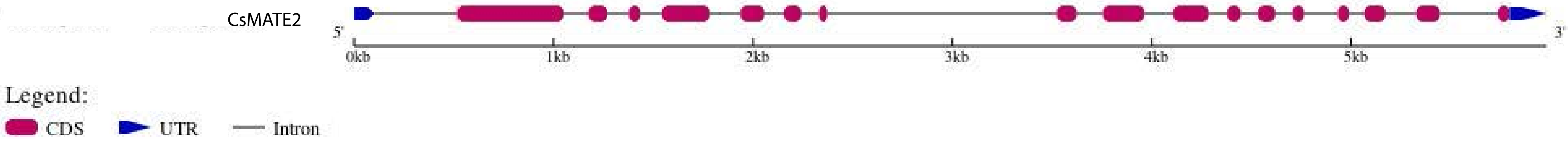
CsMATE2
5'
3'
0kb
1kb
2kb
3kb
4kb
5kb
Legend:
CDS
UTR
Intron

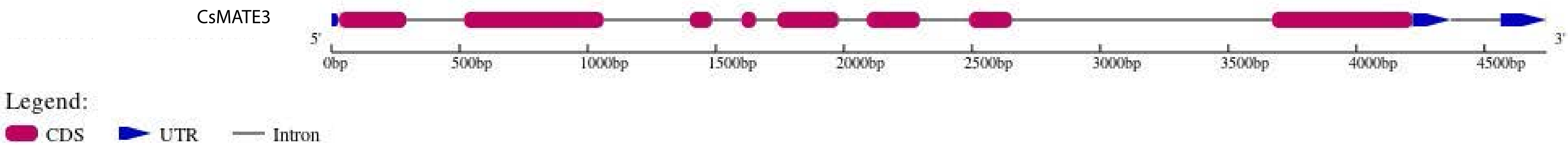
CsMATE3
5'
3'
0bp
500bp
1000bp
1500bp
2000bp
2500bp
3000bp
3500bp
4000bp
4500bp
Legend:
CDS
UTR
Intron

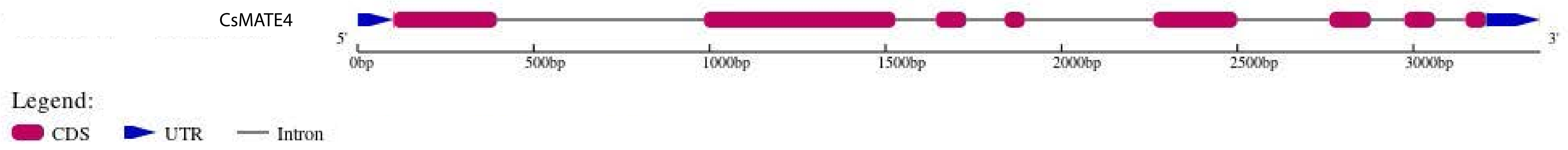
CsMATE4
5'
3'
0bp
500bp
1000bp
1500bp
2000bp
2500bp
3000bp
Legend:
CDS
UTR
Intron

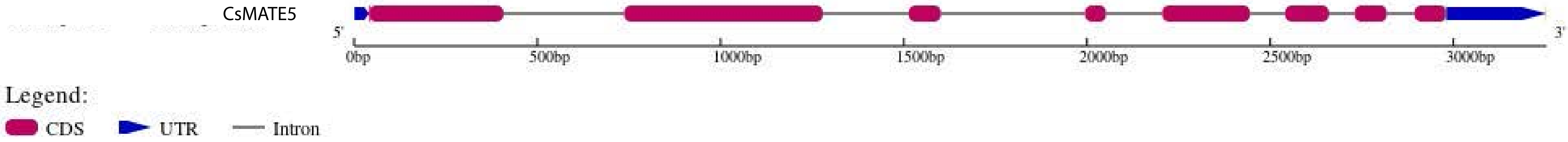

CsMATE5
5'
3'
0bp
500bp
1000bp
1500bp
2000bp
2500bp
3000bp
Legend:
CDS
UTR
Intron

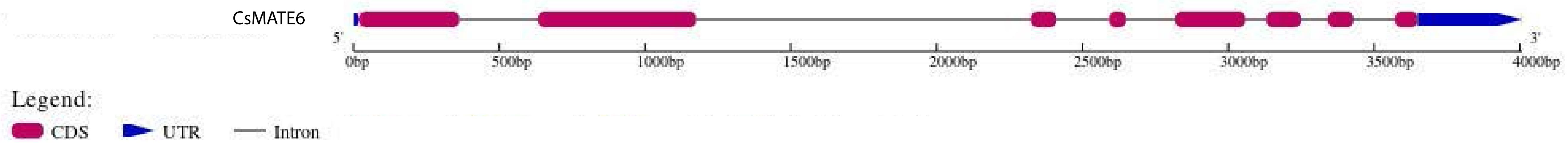
CsMATE6
5'
3'
0bp
500bp
1000bp
1500bp
2000bp
2500bp
3000bp
3500bp
4000bp
Legend:
CDS
UTR
Intron

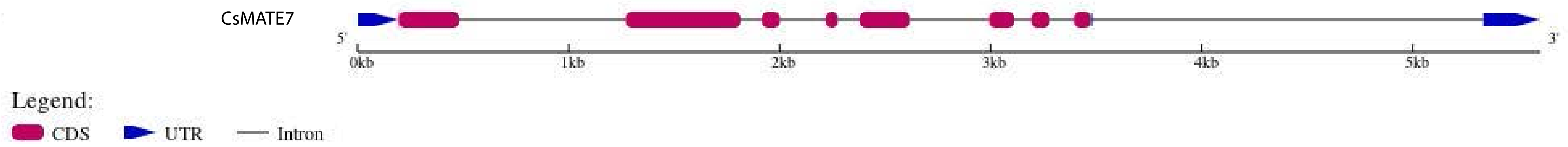
CsMATE7
5'
3'
0kb
1kb
2kb
3kb
4kb
5kb
Legend:
CDS
UTR
Intron

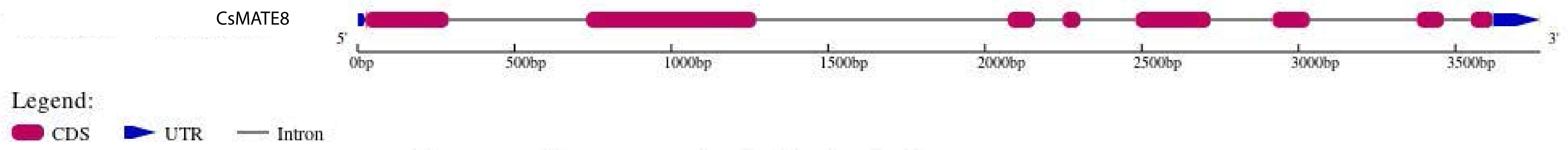

CsMATE8
5'
3'
0bp
500bp
1000bp
1500bp
2000bp
2500bp
3000bp
3500bp
Legend:
CDS
UTR
Intron

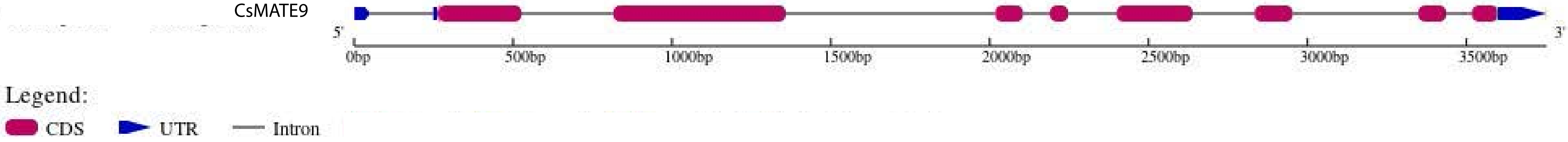
CsMATE9
5'
3'
0bp
500bp
1000bp
1500bp
2000bp
2500bp
3000bp
3500bp
Legend:
CDS
UTR
Intron

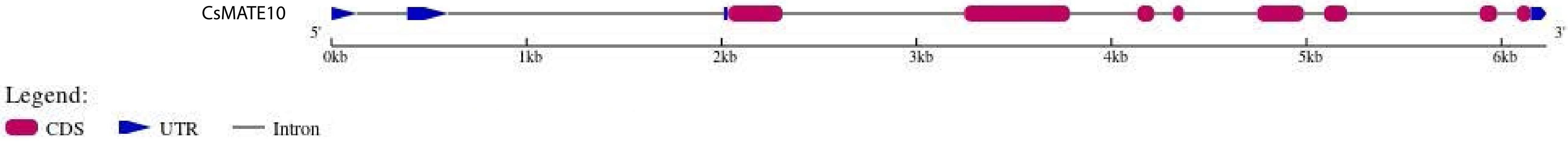
CsMATE10
5'
3'
0kb
1kb
2kb
3kb
4kb
5kb
6kb
Legend:
CDS
UTR
Intron

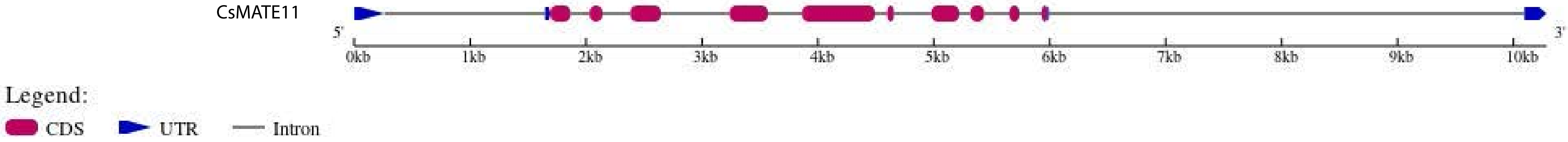
CsMATE11
5'
3'
0kb
1kb
2kb
3kb
4kb
5kb
6kb
7kb
8kb
9kb
10kb
Legend:
CDS
UTR
Intron

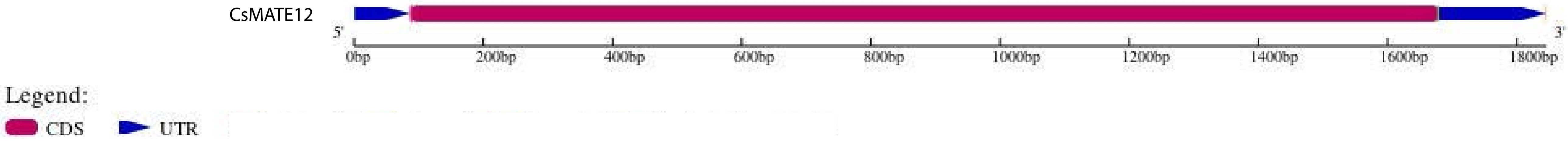
CsMATE12
5'
3'
0bp
200bp
400bp
600bp
800bp
1000bp
1200bp
1400bp
1600bp
1800bp
Legend:
CDS
UTR

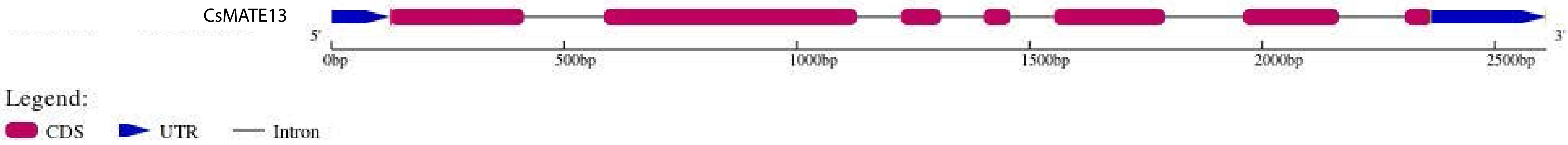
CsMATE13
5'
3'
0bp
500bp
1000bp
1500bp
2000bp
2500bp
Legend:
CDS
UTR
Intron

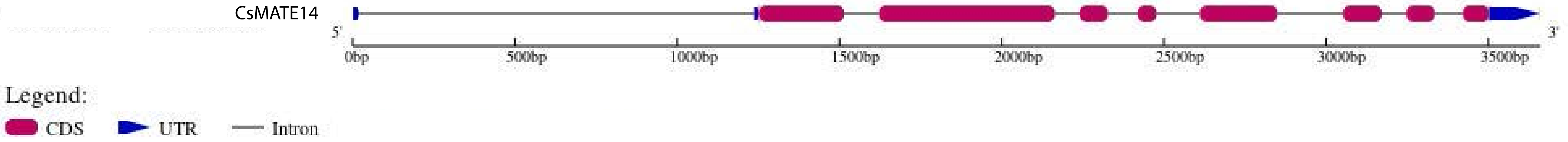
CsMATE14
5'
3'
0bp
500bp
1000bp
1500bp
2000bp
2500bp
3000bp
3500bp
Legend:
CDS
UTR
Intron

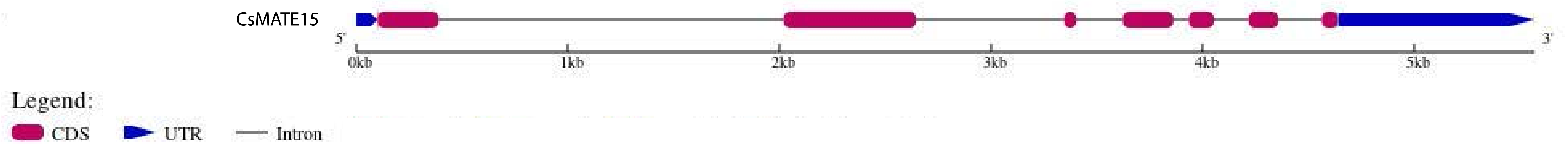
CsMATE15
5'
3'
0kb
1kb
2kb
3kb
4kb
5kb
Legend:
CDS
UTR
Intron

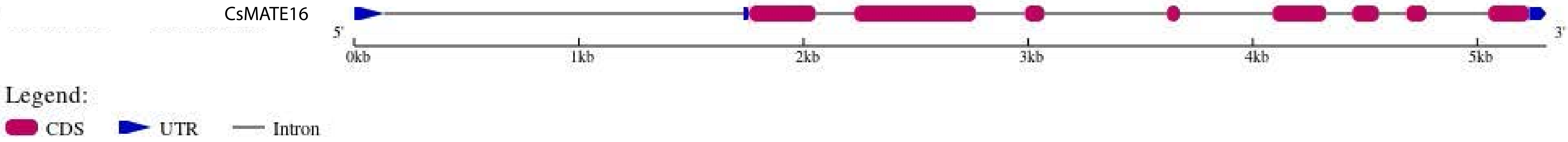
CsMATE16
5'
3'
0kb
1kb
2kb
3kb
4kb
5kb
Legend:
CDS
UTR
Intron

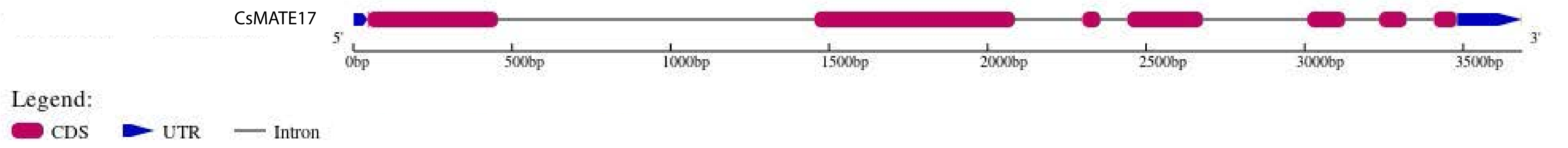
CsMATE17
5'
3'
0bp
500bp
1000bp
1500bp
2000bp
2500bp
3000bp
3500bp
Legend:
CDS
UTR
Intron

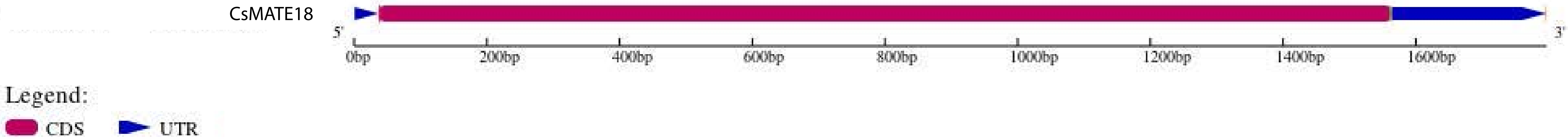
CsMATE18
5'
3'
0bp
200bp
400bp
600bp
800bp
1000bp
1200bp
1400bp
1600bp
Legend:
CDS
UTR

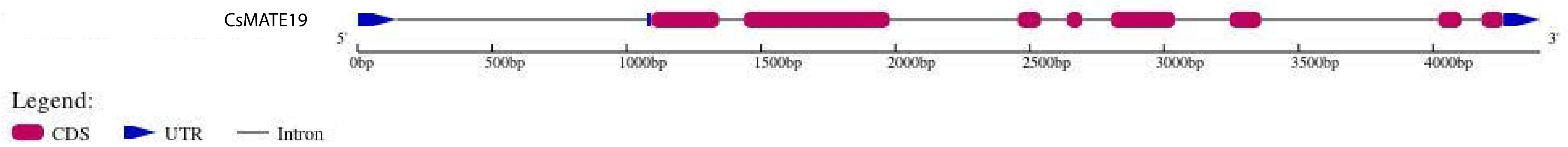
CsMATE19
5'
3'
0bp
500bp
1000bp
1500bp
2000bp
2500bp
3000bp
3500bp
4000bp
Legend:
CDS
UTR
Intron

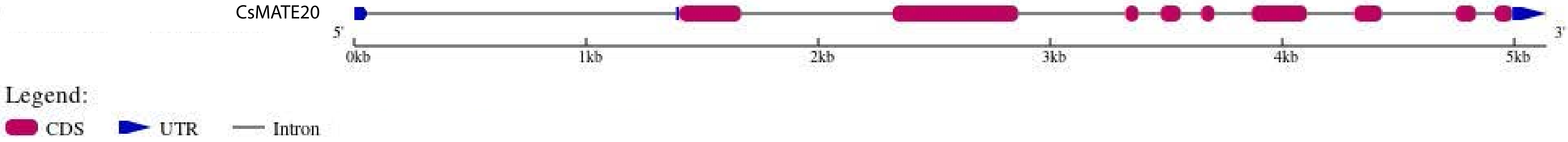
CsMATE20
5'
3'
0kb
1kb
2kb
3kb
4kb
5kb
Legend:
CDS
UTR
Intron

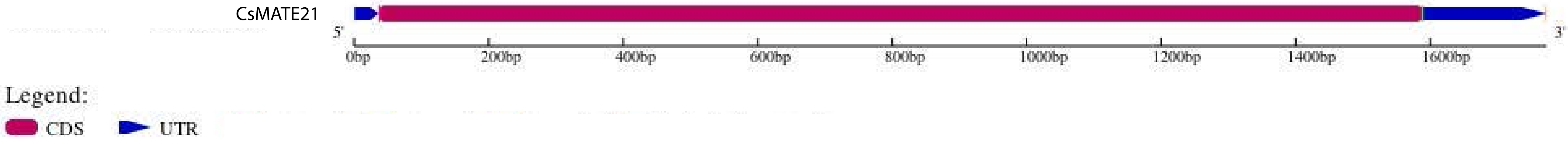
CsMATE21
5'
3'
0bp
200bp
400bp
600bp
800bp
1000bp
1200bp
1400bp
1600bp
Legend:
CDS
UTR

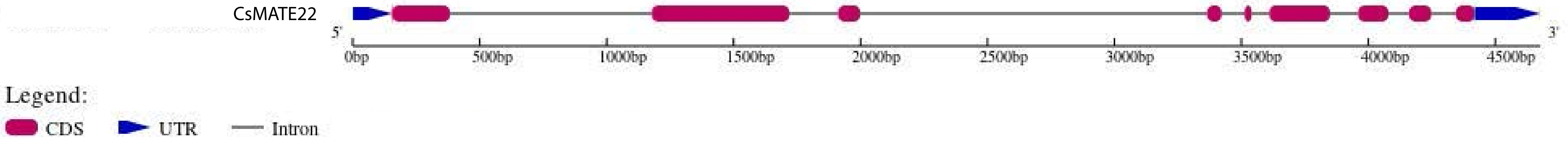
CsMATE22
5'
3'
0bp
500bp
1000bp
1500bp
2000bp
2500bp
3000bp
3500bp
4000bp
4500bp
Legend:
CDS
UTR
Intron

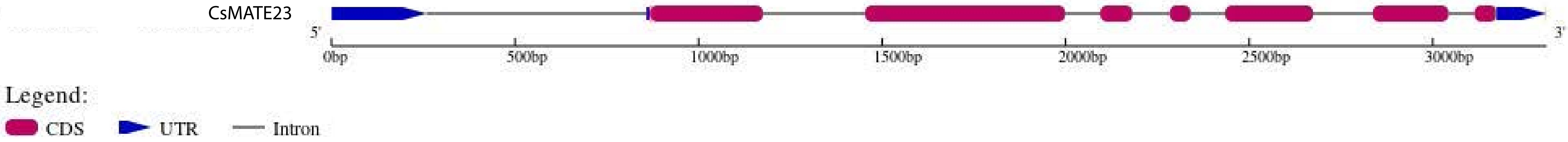
CsMATE23
5'
3'
0bp
500bp
1000bp
1500bp
2000bp
2500bp
3000bp
Legend:
CDS
UTR
Intron

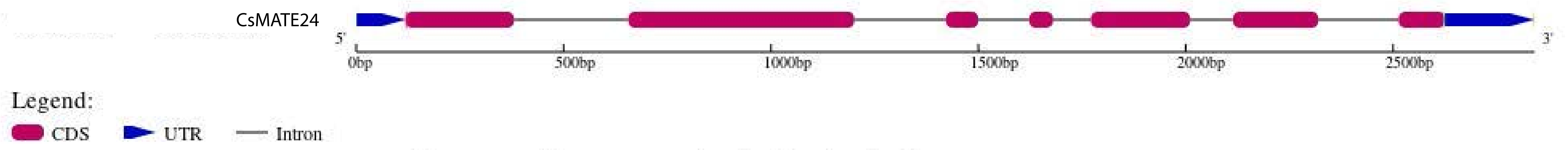
CsMATE24
5'
3'
0bp
500bp
1000bp
1500bp
2000bp
2500bp
Legend:
CDS
UTR
Intron

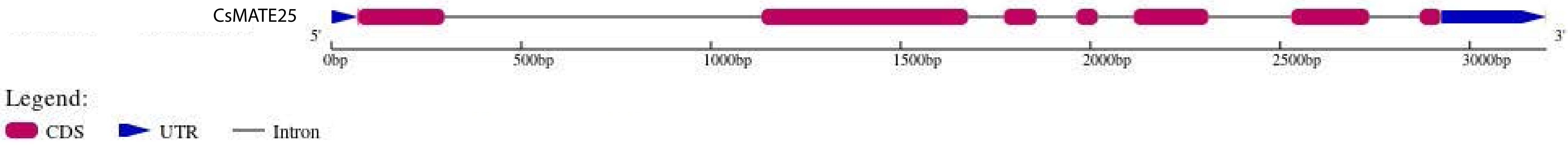
CsMATE25
5'
3'
0bp
500bp
1000bp
1500bp
2000bp
2500bp
3000bp
Legend:
CDS
UTR
Intron

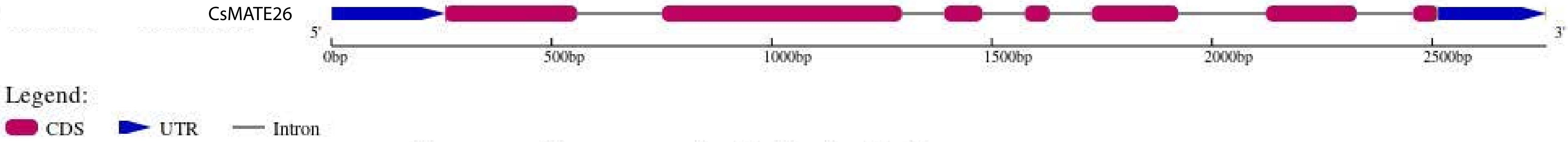
CsMATE26
5'
3'
0bp
500bp
1000bp
1500bp
2000bp
2500bp
Legend:
CDS
UTR
Intron

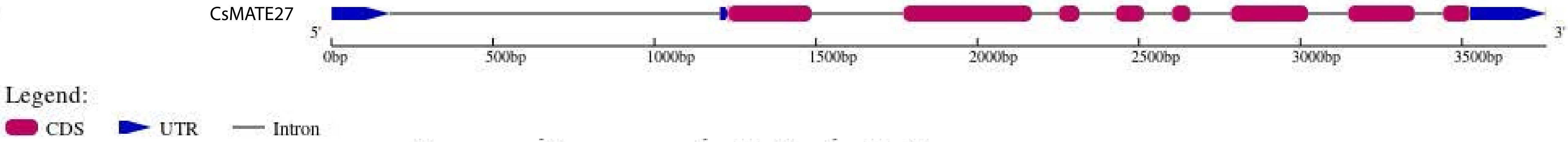
CsMATE27
5'
3'
0bp
500bp
1000bp
1500bp
2000bp
2500bp
3000bp
3500bp
Legend:
CDS
UTR
Intron

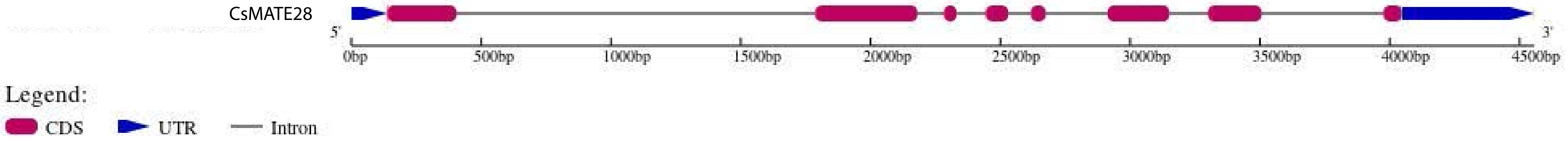
CsMATE28
5'
3'
0bp
500bp
1000bp
1500bp
2000bp
2500bp
3000bp
3500bp
4000bp
4500bp
Legend:
CDS
UTR
Intron

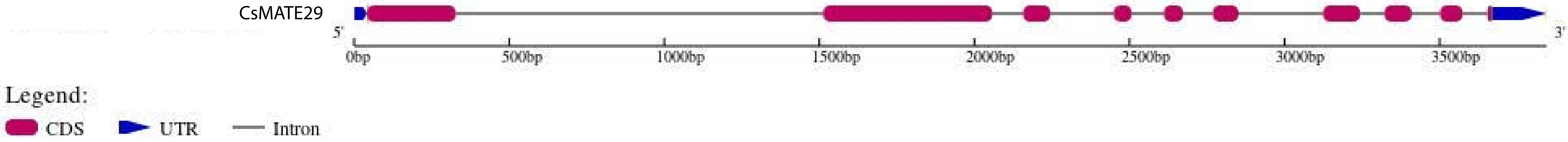
CsMATE29
5'
3'
0bp
500bp
1000bp
1500bp
2000bp
2500bp
3000bp
3500bp
Legend:
CDS
UTR
Intron

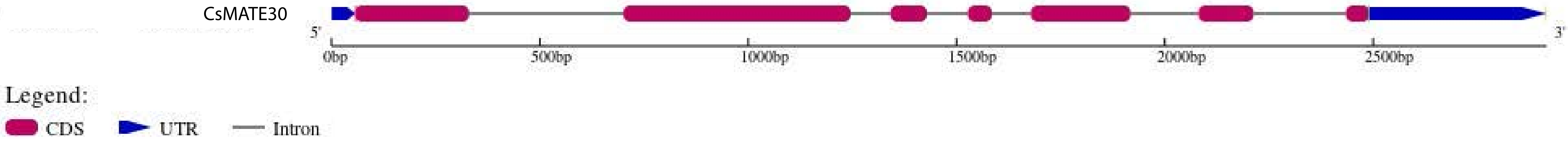
CsMATE30
5'
3'
0bp
500bp
1000bp
1500bp
2000bp
2500bp
Legend:
CDS
UTR
Intron

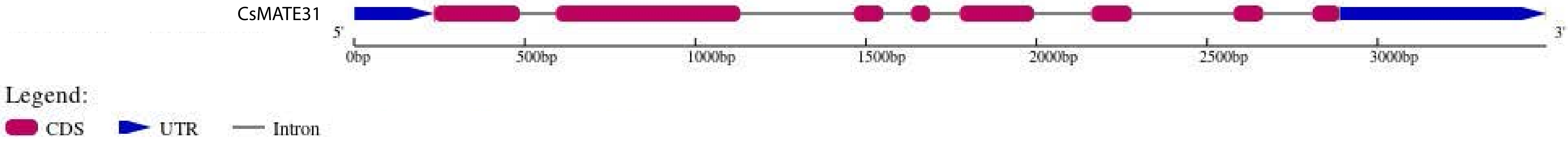
CsMATE31
5'
3'
0bp
500bp
1000bp
1500bp
2000bp
2500bp
3000bp
Legend:
CDS
UTR
Intron

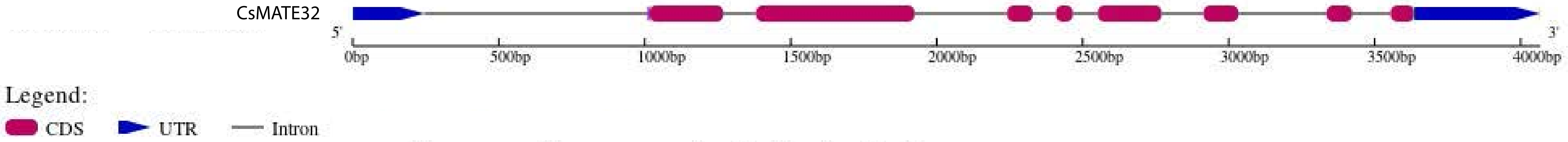
CsMATE32
5'
3'
0bp
500bp
1000bp
1500bp
2000bp
2500bp
3000bp
3500bp
4000bp
Legend:
CDS
UTR
Intron

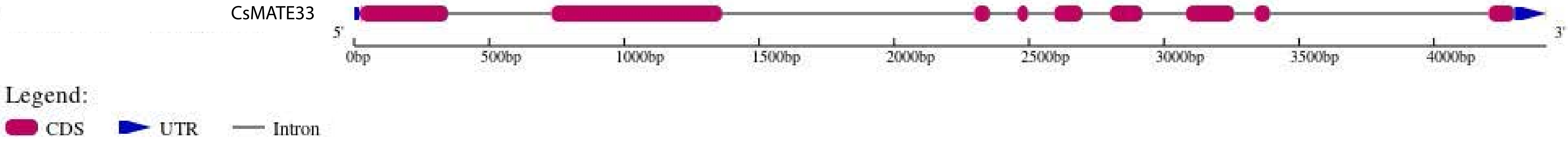
CsMATE33
5'
3'
0bp
500bp
1000bp
1500bp
2000bp
2500bp
3000bp
3500bp
4000bp
Legend:
CDS
UTR
Intron

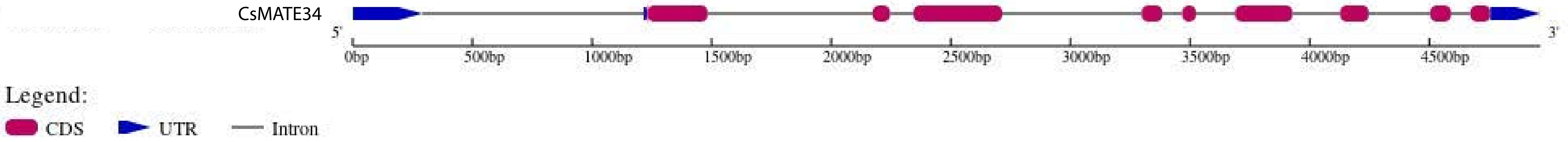
CsMATE34
5'
3'
0bp
500bp
1000bp
1500bp
2000bp
2500bp
3000bp
3500bp
4000bp
4500bp
Legend:
CDS
UTR
Intron

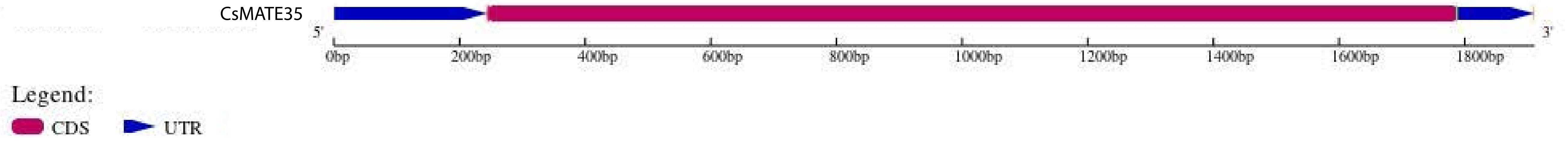

CsMATE35
5'
3'
0bp
200bp
400bp
600bp
800bp
1000bp
1200bp
1400bp
1600bp
1800bp
Legend:
CDS
UTR

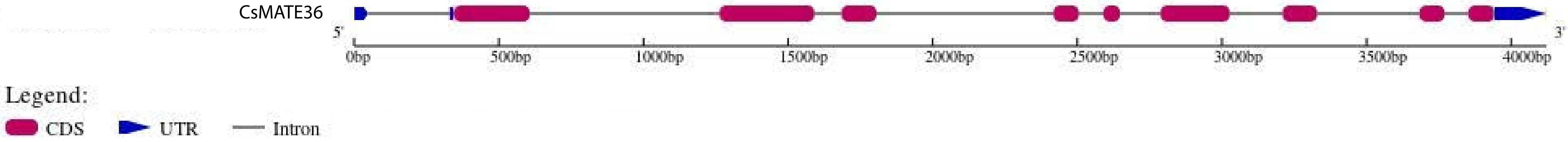
CsMATE36
5'
3'
0bp
500bp
1000bp
1500bp
2000bp
2500bp
3000bp
3500bp
4000bp
Legend:
CDS
UTR
Intron

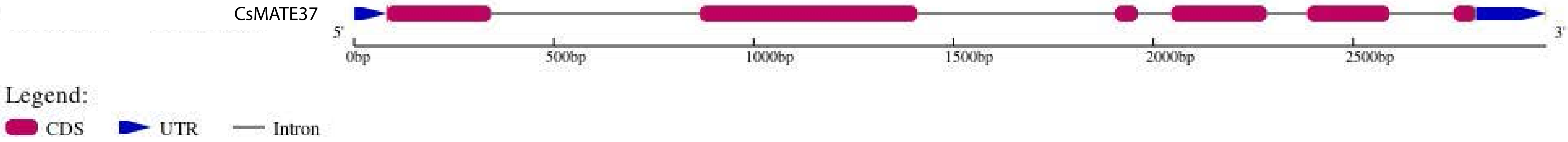
CsMATE37
5'
3'
0bp
500bp
1000bp
1500bp
2000bp
2500bp
Legend:
CDS
UTR
Intron

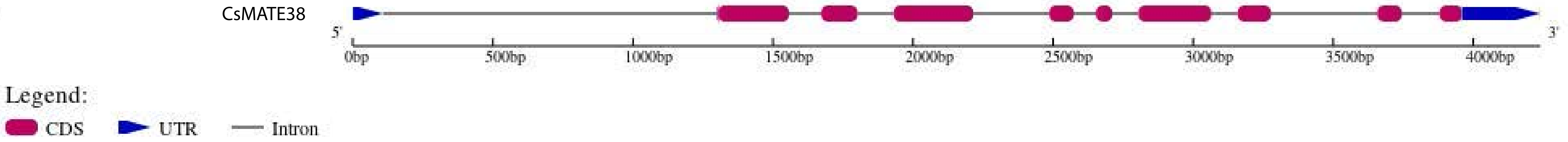
CsMATE38
5'
3'
0bp
500bp
1000bp
1500bp
2000bp
2500bp
3000bp
3500bp
4000bp
Legend:
CDS
UTR
Intron

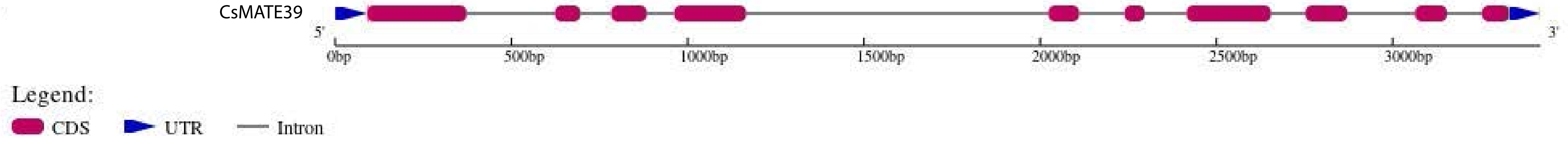
CsMATE39
5'
3'
0bp
500bp
1000bp
1500bp
2000bp
2500bp
3000bp
Legend:
CDS
UTR
Intron

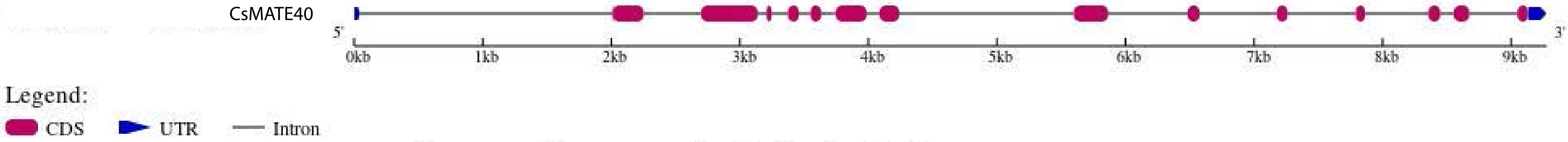
CsMATE40
5'
3'
0kb
1kb
2kb
3kb
4kb
5kb
6kb
7kb
8kb
9kb
Legend:
CDS
UTR
Intron

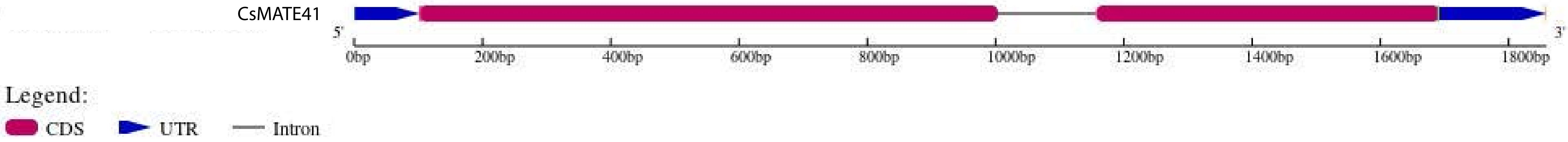
CsMATE41
5'
3'
0bp
200bp
400bp
600bp
800bp
1000bp
1200bp
1400bp
1600bp
1800bp
Legend:
CDS
UTR
Intron

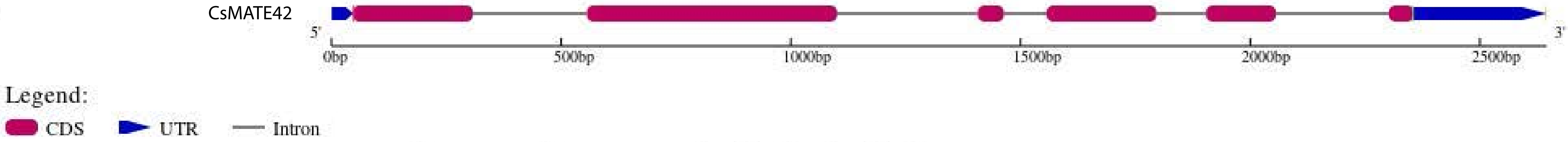
CsMATE42
5'
3'
0bp
500bp
1000bp
1500bp
2000bp
2500bp
Legend:
CDS
UTR
Intron

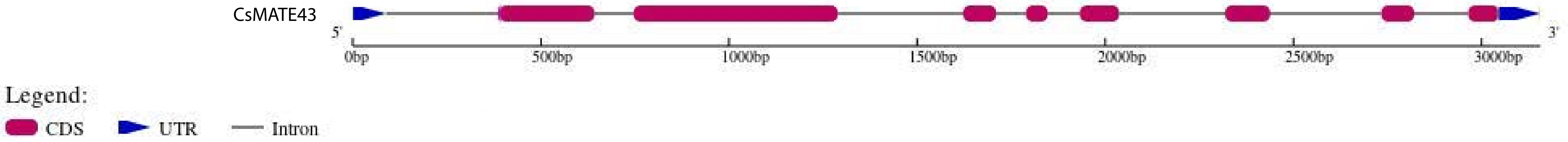
CsMATE43
5'
3'
0bp
500bp
1000bp
1500bp
2000bp
2500bp
3000bp
Legend:
CDS
UTR
Intron

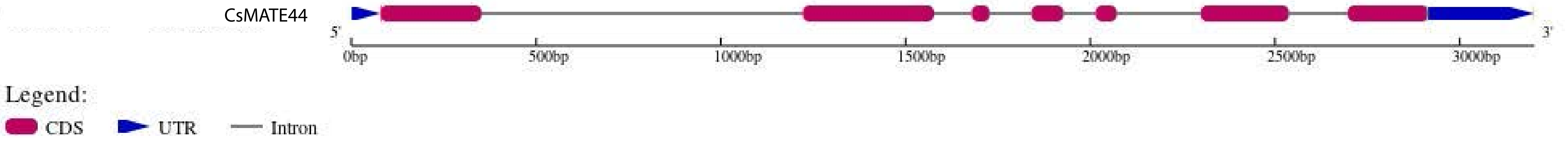
CsMATE44
5'
3'
0bp
500bp
1000bp
1500bp
2000bp
2500bp
3000bp
Legend:
CDS
UTR
Intron

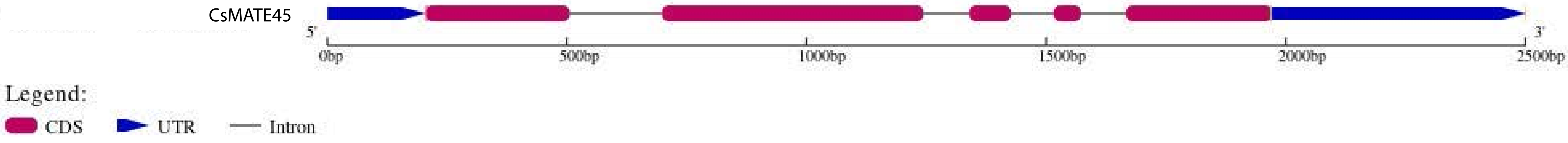

CsMATE45
5'
3'
0bp
500bp
1000bp
1500bp
2000bp
2500bp
Legend:
CDS
UTR
Intron

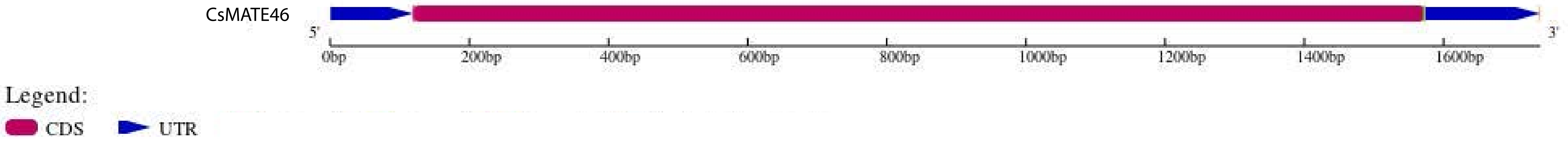

CsMATE46
5'
3'
0bp
200bp
400bp
600bp
800bp
1000bp
1200bp
1400bp
1600bp
Legend:
CDS
UTR

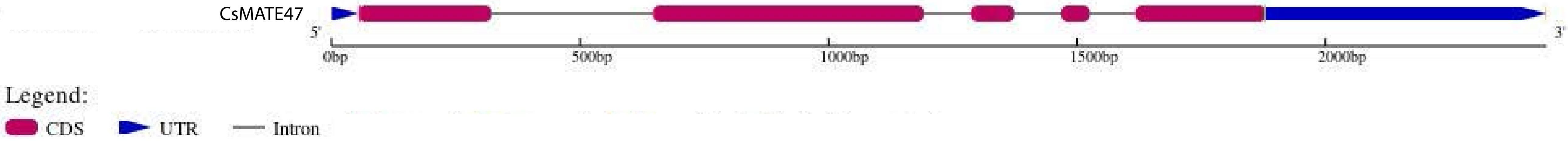
CsMATE47
5'
3'
0bp
500bp
1000bp
1500bp
2000bp
Legend:
CDS
UTR
Intron

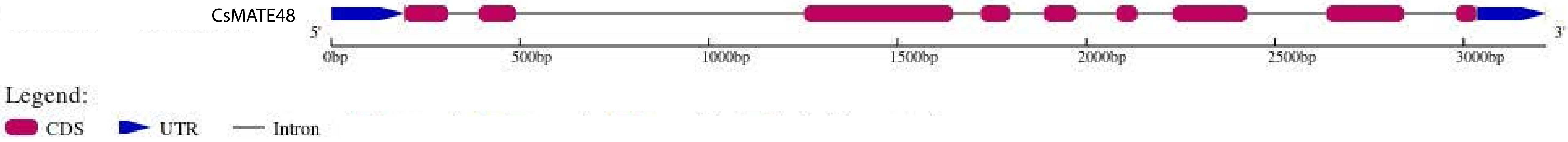
CsMATE48
5'
3'
0bp
500bp
1000bp
1500bp
2000bp
2500bp
3000bp
Legend:
CDS
UTR
Intron

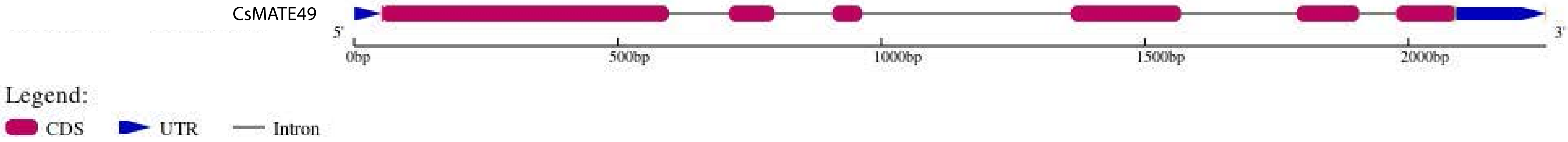
CsMATE49
5'
3'
0bp
500bp
1000bp
1500bp
2000bp
Legend:
CDS
UTR
Intron

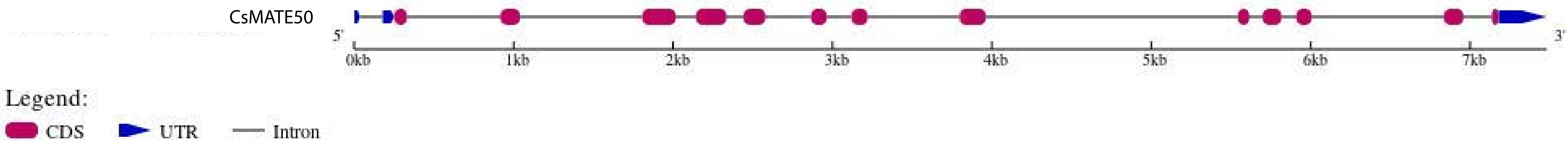
CsMATE50
5'
3'
0kb
1kb
2kb
3kb
4kb
5kb
6kb
7kb
Legend:
CDS
UTR
Intron

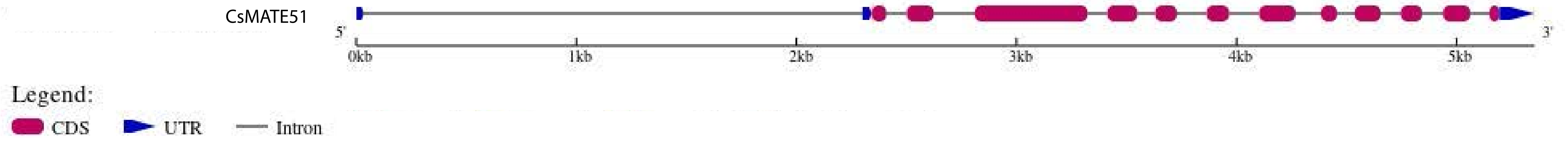
CsMATE51
5'
3'
0kb
1kb
2kb
3kb
4kb
5kb
Legend:
CDS
UTR
Intron

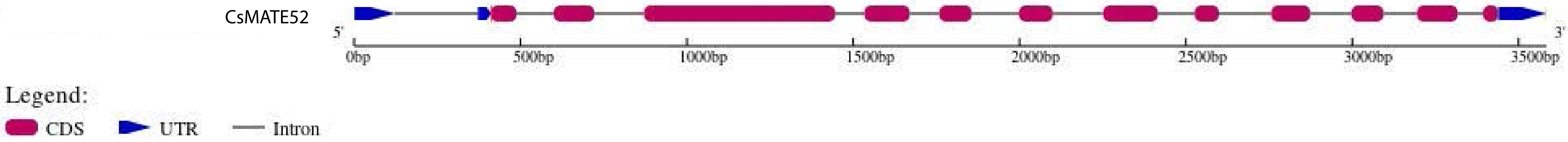
CsMATE52
5'
3'
0bp
500bp
1000bp
1500bp
2000bp
2500bp
3000bp
3500bp
Legend:
CDS
UTR
Intron

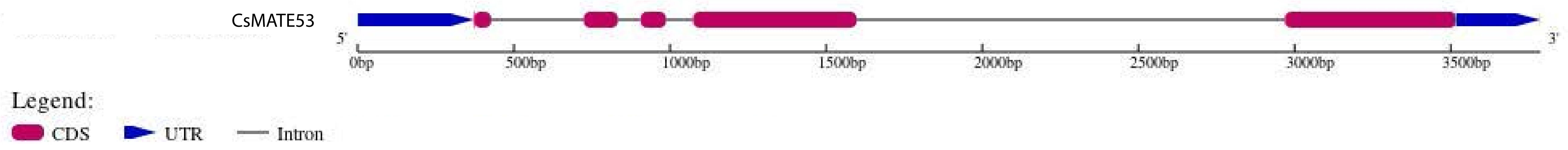
CsMATE53
5'
3'
0bp
500bp
1000bp
1500bp
2000bp
2500bp
3000bp
3500bp
Legend:
CDS
UTR
Intron

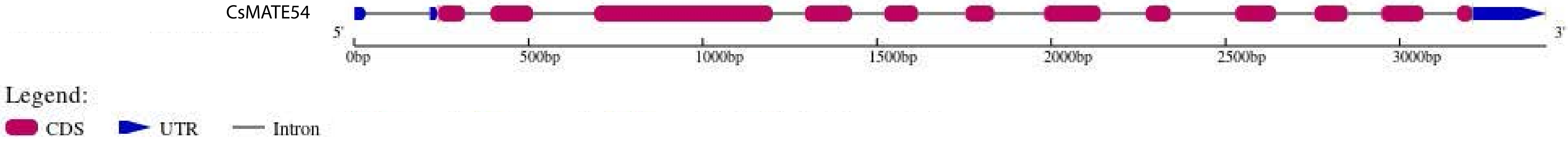
CsMATE54
5'
3'
0bp
500bp
1000bp
1500bp
2000bp
2500bp
3000bp
Legend:
CDS
UTR
Intron

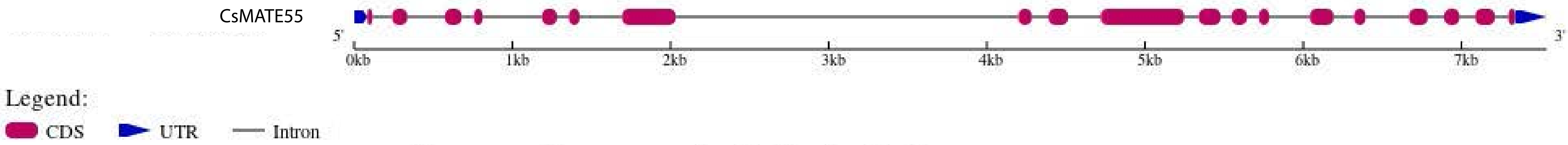
CsMATE55
5'
3'
0kb
1kb
2kb
3kb
4kb
5kb
6kb
7kb
Legend:
CDS
UTR
Intron

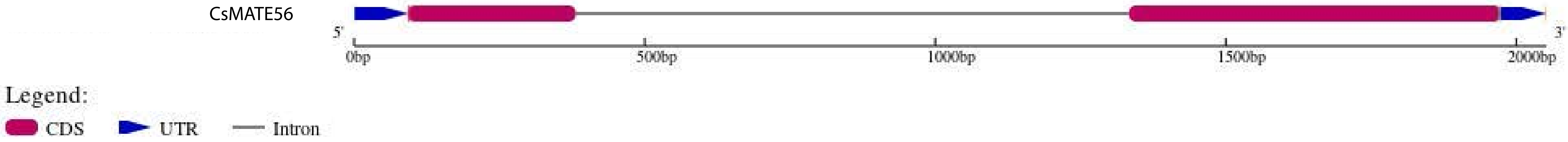
CsMATE56
5'
3'
0bp
500bp
1000bp
1500bp
2000bp
Legend:
CDS
UTR
Intron

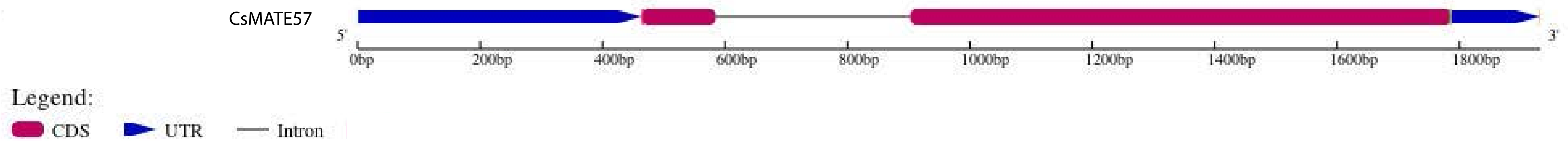

CsMATE57
5'
3'
0bp
200bp
400bp
600bp
800bp
1000bp
1200bp
1400bp
1600bp
1800bp
Legend:
CDS
UTR
Intron

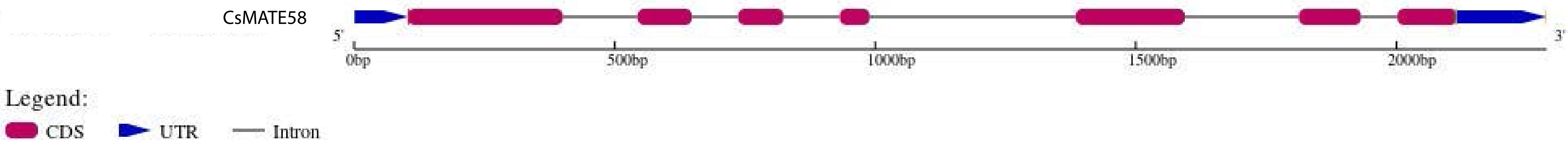

CsMATE58
5'
3'
0bp
500bp
1000bp
1500bp
2000bp
Legend:
CDS
UTR
Intron

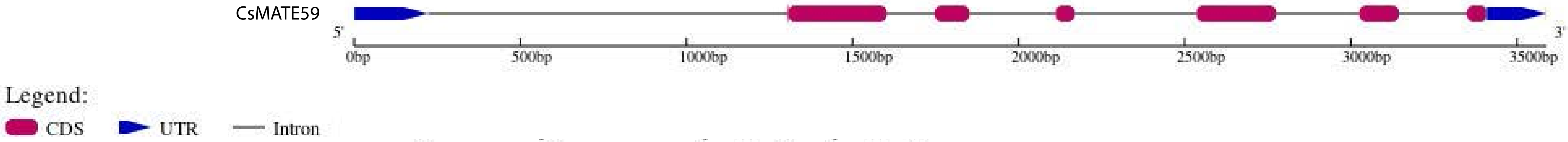
CsMATE59
5'
3'
0bp
500bp
1000bp
1500bp
2000bp
2500bp
3000bp
3500bp
Legend:
CDS
UTR
Intron

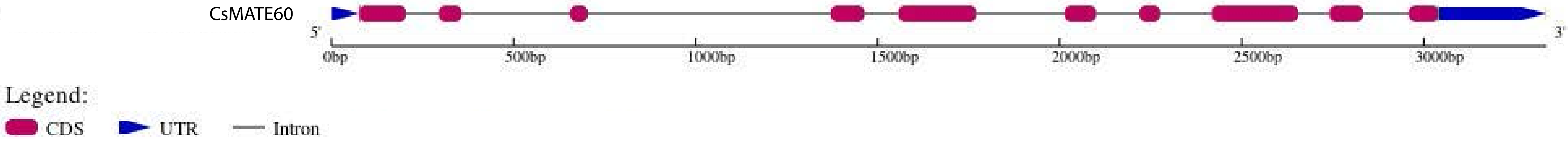
CsMATE60
5'
3'
0bp
500bp
1000bp
1500bp
2000bp
2500bp
3000bp
Legend:
CDS
UTR
Intron

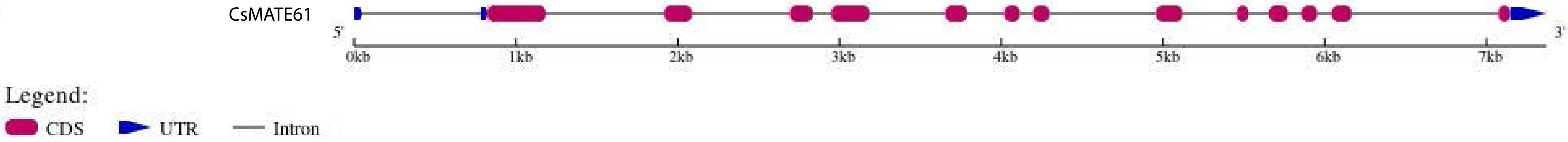
CsMATE61
5'
3'
0kb
1kb
2kb
3kb
4kb
5kb
6kb
7kb
Legend:
CDS
UTR
Intron

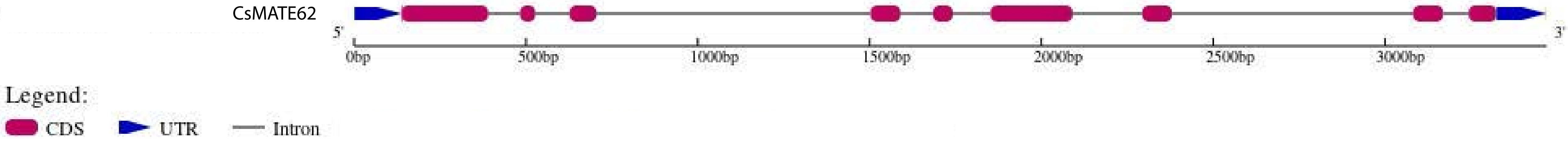

CsMATE62
5'
3'
0bp
500bp
1000bp
1500bp
2000bp
2500bp
3000bp
Legend:
CDS
UTR
Intron

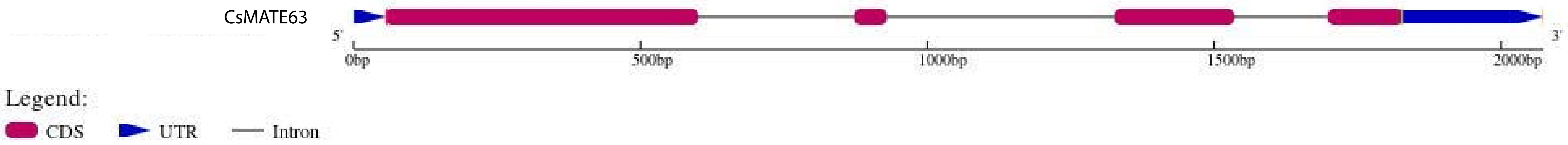
CsMATE63
5'
3'
0bp
500bp
1000bp
1500bp
2000bp
Legend:
CDS
UTR
Intron

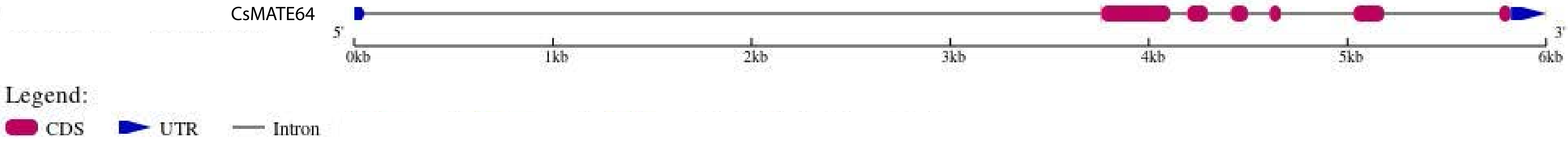

CsMATE64
5'
3'
0kb
1kb
2kb
3kb
4kb
5kb
6kb
Legend:
CDS
UTR
Intron

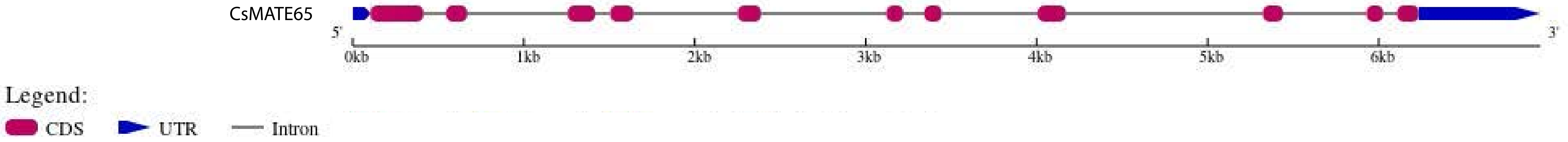
CsMATE65
5'
3'
0kb
1kb
2kb
3kb
4kb
5kb
6kb
Legend:
CDS
UTR
Intron

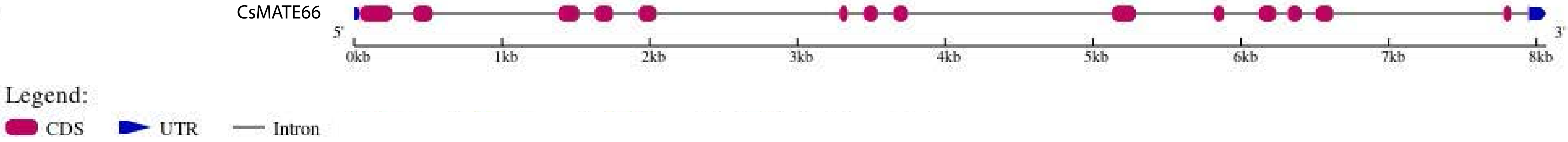
CsMATE66
5'
3'
0kb
1kb
2kb
3kb
4kb
5kb
6kb
7kb
8kb
Legend:
CDS
UTR
Intron

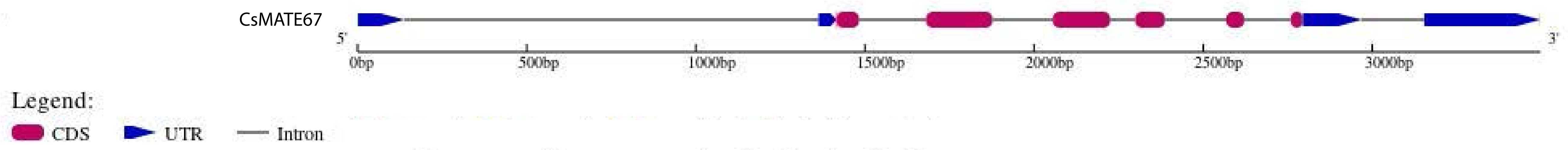

CsMATE67
5'
3'
0bp
500bp
1000bp
1500bp
2000bp
2500bp
3000bp
Legend:
CDS
UTR
Intron

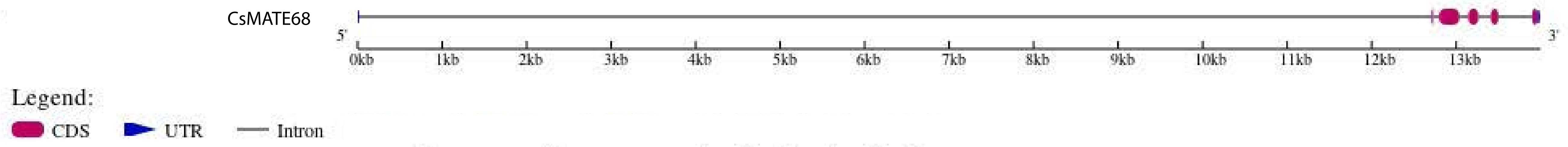
CsMATE68
5'
3'
0kb
1kb
2kb
3kb
4kb
5kb
6kb
7kb
8kb
9kb
10kb
11kb
12kb
13kb
Legend:
CDS
UTR
Intron

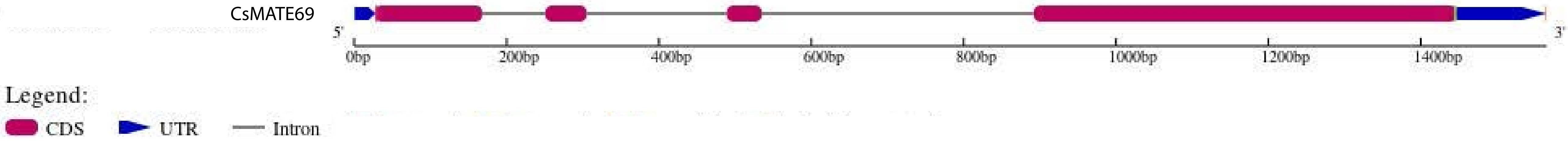
CsMATE69
5'
3'
0bp
200bp
400bp
600bp
800bp
1000bp
1200bp
1400bp
Legend:
CDS
UTR
Intron

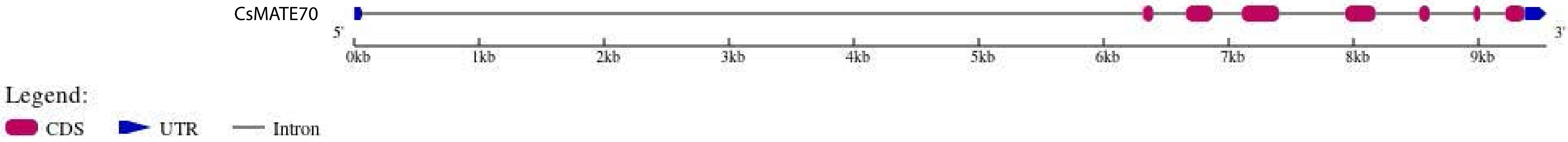
CsMATE70
5'
3'
0kb
1kb
2kb
3kb
4kb
5kb
6kb
7kb
8kb
9kb
Legend:
CDS
UTR
Intron

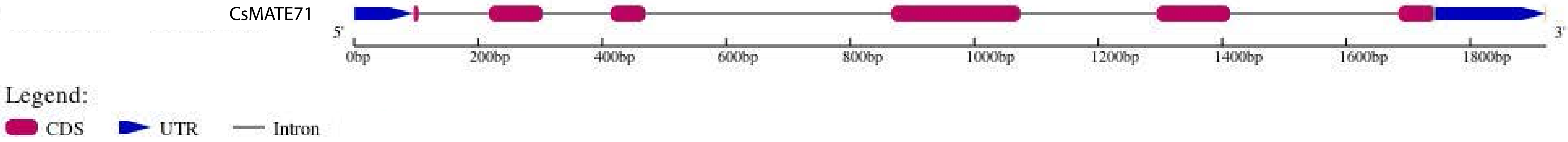
CsMATE71
5'
3'
0bp
200bp
400bp
600bp
800bp
1000bp
1200bp
1400bp
1600bp
1800bp
Legend:
CDS
UTR
Intron

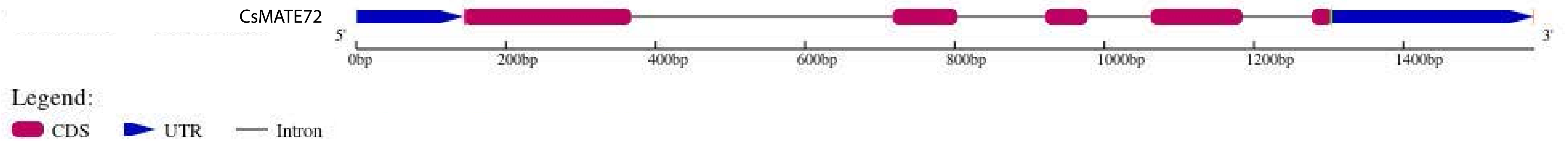
CsMATE72
5'
3'
0bp
200bp
400bp
600bp
800bp
1000bp
1200bp
1400bp
Legend:
CDS
UTR
Intron

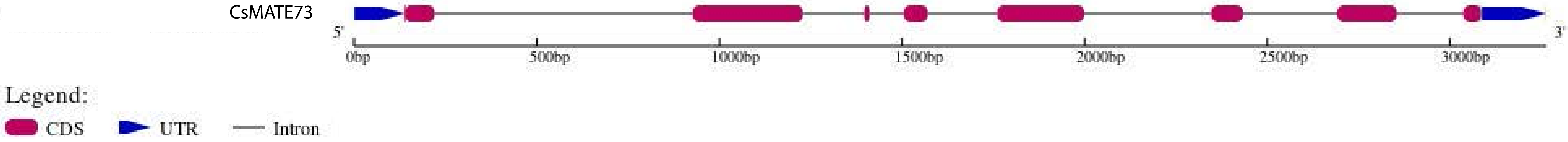
CsMATE73
5'
3'
0bp
500bp
1000bp
1500bp
2000bp
2500bp
3000bp
Legend:
CDS
UTR
Intron

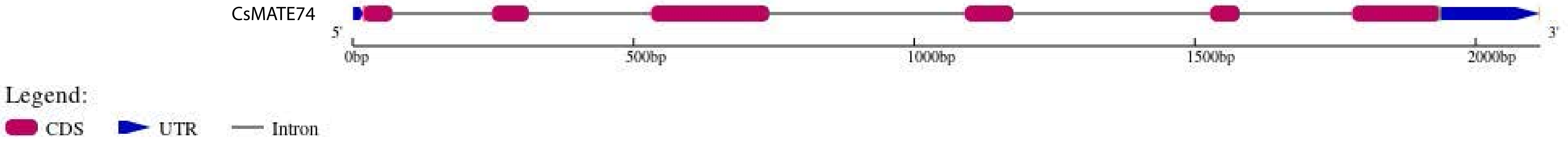
CsMATE74
5'
3'
0bp
500bp
1000bp
1500bp
2000bp
Legend:
CDS
UTR
Intron

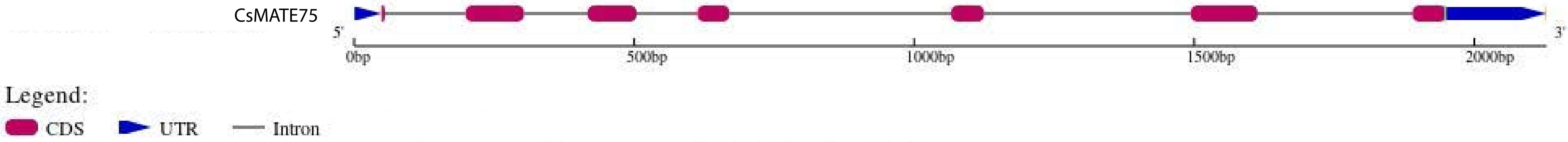
CsMATE75
5'
3'
0bp
500bp
1000bp
1500bp
2000bp
Legend:
CDS
UTR
Intron

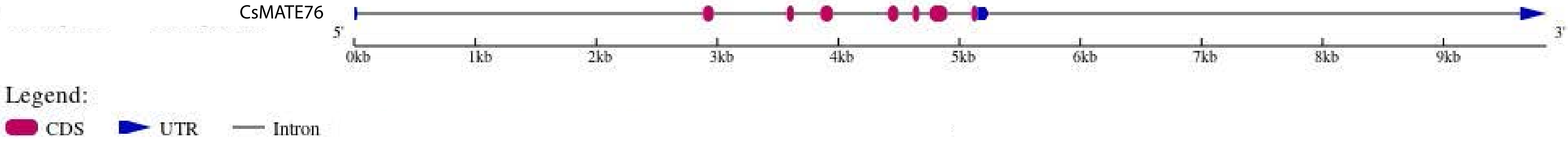
CsMATE76
5'
3'
0kb
1kb
2kb
3kb
4kb
5kb
6kb
7kb
8kb
9kb
Legend:
CDS
UTR
Intron

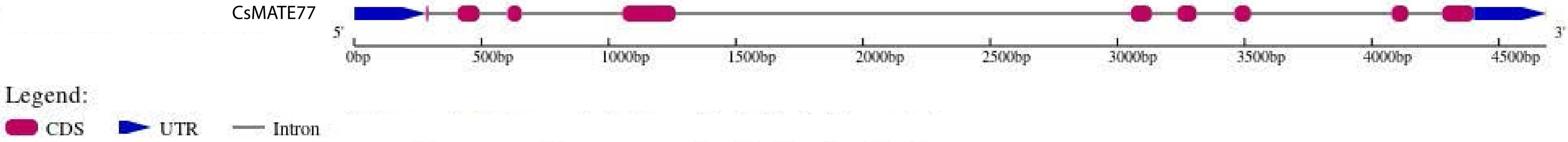
CsMATE77
5'
3'
0bp
500bp
1000bp
1500bp
2000bp
2500bp
3000bp
3500bp
4000bp
4500bp
Legend:
CDS
UTR
Intron

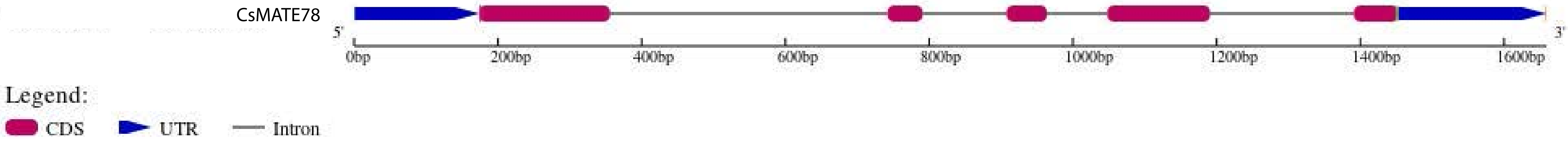
CsMATE78
5'
3'
0bp
200bp
400bp
600bp
800bp
1000bp
1200bp
1400bp
1600bp
Legend:
CDS
UTR
Intron

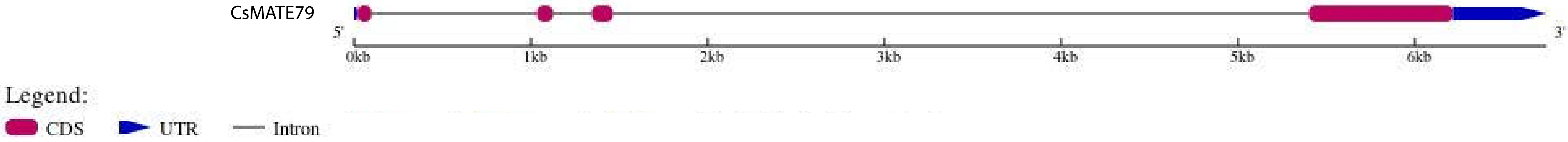
CsMATE79
5'
3'
0kb
1kb
2kb
3kb
4kb
5kb
6kb
Legend:
CDS
UTR
Intron

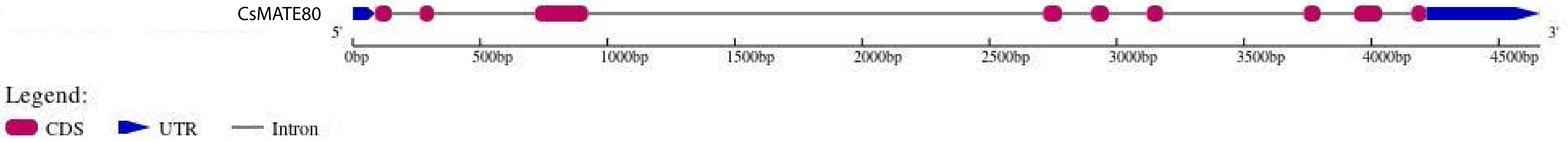
CsMATE80
5'
3'
0bp
500bp
1000bp
1500bp
2000bp
2500bp
3000bp
3500bp
4000bp
4500bp
Legend:
CDS
UTR
Intron

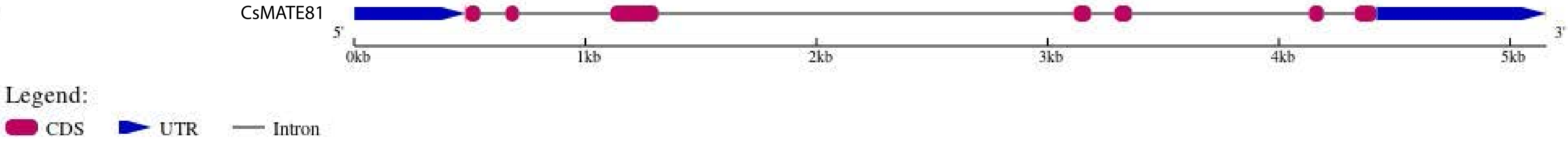
CsMATE81
5'
3'
0kb
1kb
2kb
3kb
4kb
5kb
Legend:
CDS
UTR
Intron

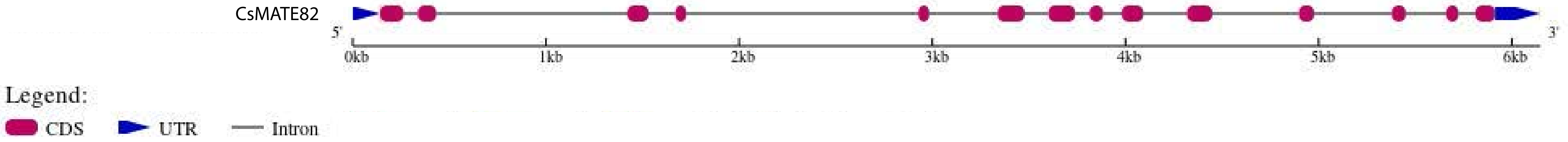
CsMATE82
5'
3'
0kb
1kb
2kb
3kb
4kb
5kb
6kb
Legend:
CDS
UTR
Intron

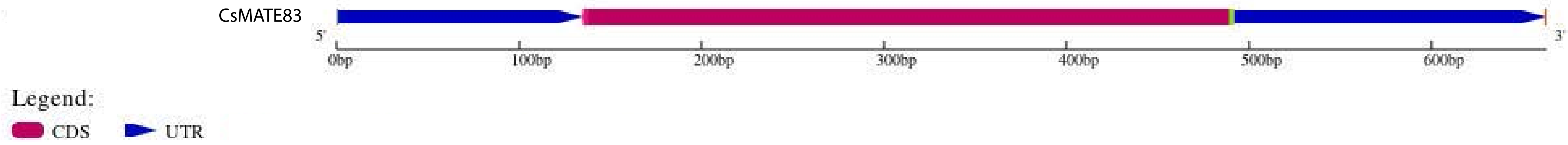

CsMATE83
5'
3'
0bp
100bp
200bp
300bp
400bp
500bp
600bp
Legend:
CDS
UTR

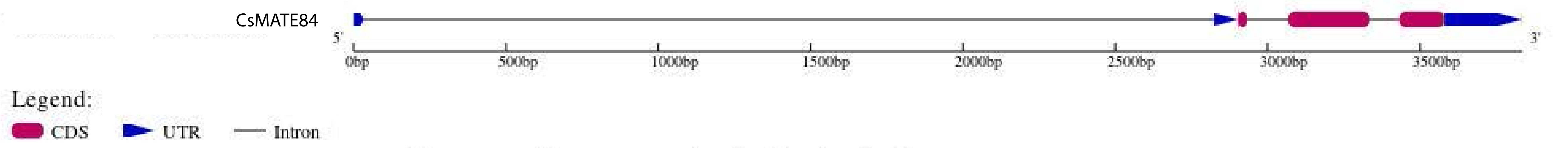
CsMATE84
5'
3'
0bp
500bp
1000bp
1500bp
2000bp
2500bp
3000bp
3500bp
Legend:
CDS
UTR
Intron

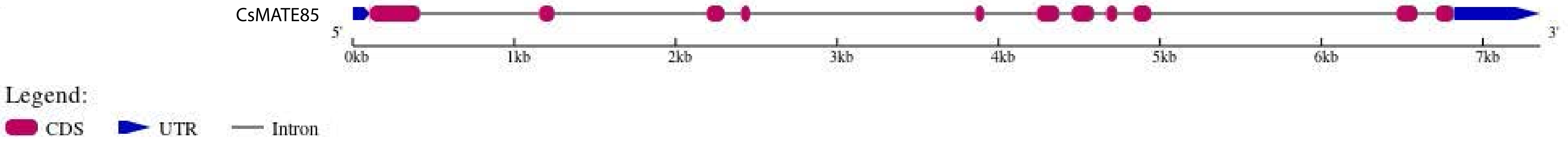
CsMATE85
5'
3'
0kb
1kb
2kb
3kb
4kb
5kb
6kb
7kb
Legend:
CDS
UTR
Intron

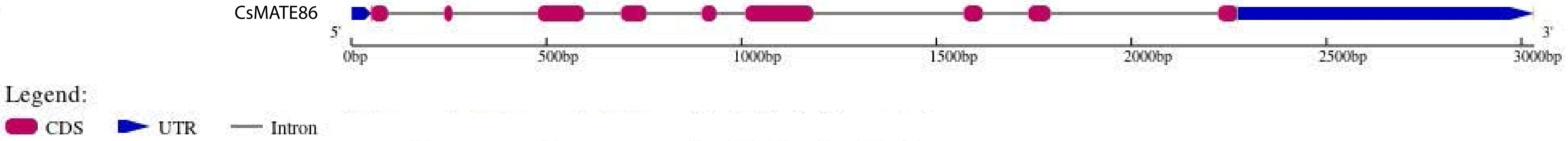
CsMATE86
5'
3'
0bp
500bp
1000bp
1500bp
2000bp
2500bp
3000bp
Legend:
CDS
UTR
Intron

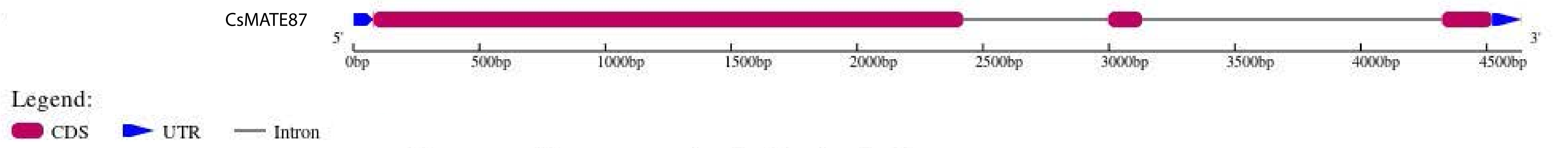
CsMATE87
5'
3'
0bp
500bp
1000bp
1500bp
2000bp
2500bp
3000bp
3500bp
4000bp
4500bp
Legend:
CDS
UTR
Intron

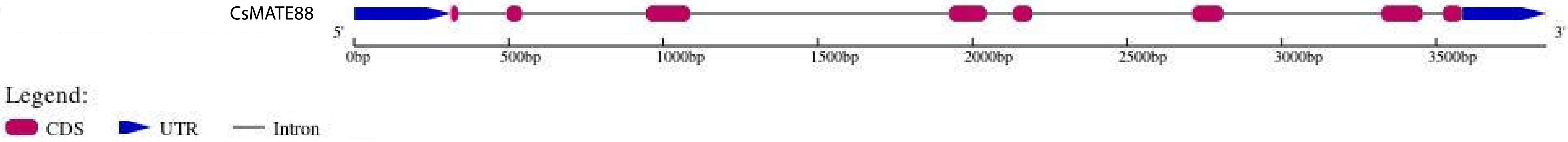
CsMATE88
5'
3'
0bp
500bp
1000bp
1500bp
2000bp
2500bp
3000bp
3500bp
Legend:
CDS
UTR
Intron

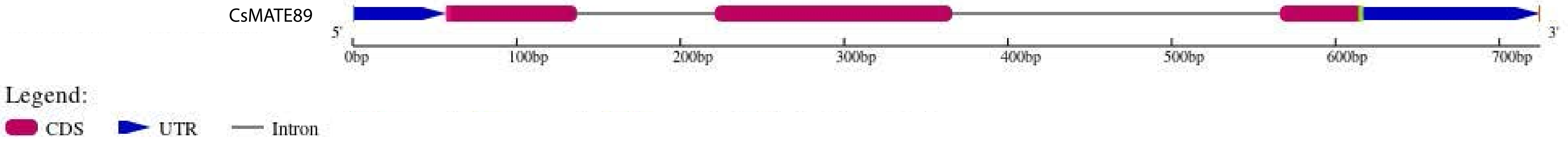
CsMATE89
5'
3'
0bp
100bp
200bp
300bp
400bp
500bp
600bp
700bp
Legend:
CDS
UTR
Intron

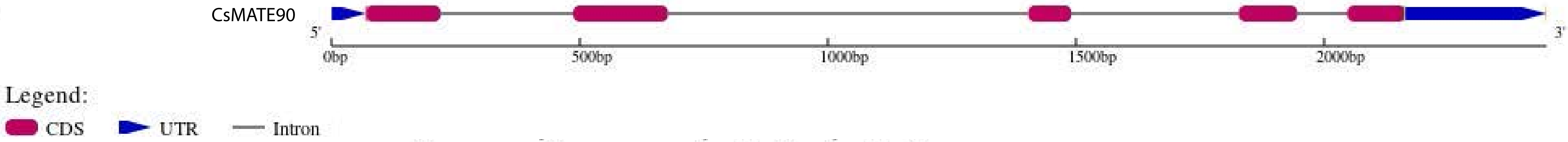
CsMATE90
5'
3'
0bp
500bp
1000bp
1500bp
2000bp
Legend:
CDS
UTR
Intron

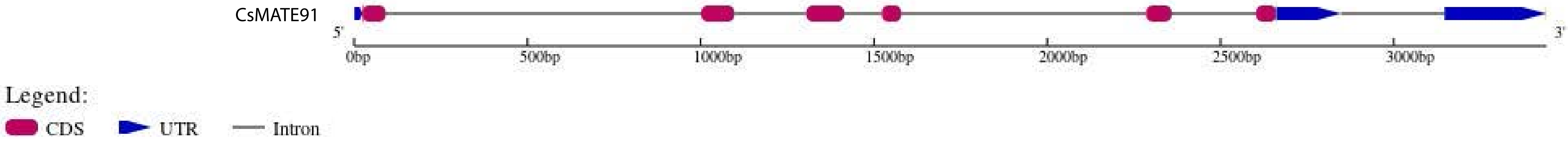
CsMATE91
5'
3'
0bp
500bp
1000bp
1500bp
2000bp
2500bp
3000bp
Legend:
CDS
UTR
Intron
