# Supplementary material for: Genome-wide mining and characterization of MATE transporters in Coriandrum sativum L
Source: Mol Biol Res Commun. 2024;13(3):155–64. doi: 10.22099/mbrc.2024.49840.1954 (PMC11194028; doi:10.22099/mbrc.2024.49840.1954)
Supplement: File S1 [file mbrc-13-155-s003.pdf]

>CsMATE1
MADQEGGMAVNTPDNEEKQMVAKSTWNEFVAETKKICLIFVPMLL
VSTSQYLLTSVSTMMVGHVGKLYLSGAVVSVSFTNVTGFSVLYGM
SSALETLCGQ
AYGAKQHKKLSAYTYGAMISLLLVCIPIAILWIFMEKLLILIHQD
PLIAHEAGKFSIRTIPALFPYAILQPLVRYLQSQYLVLPLLASSV
ATLAFHIPVC
WAFVFKFHMGSNGAALAIGLSYWFNAIFLGLYAMYSPKCADTRAR
FTMEAFSTIKDFVRIGIPSALMVCLEWWACEIIIFLAGVMKNPQL
ETSVLSISIT
IAVLHSFAPYSLSVAASIRVSNELGAGNEKAVRRSVWVVLVLGII
EVSIAAAVVLSLRYDLGRAFVSDNQIVDYLRKMTPFICLKMLLDS
IQAILSGVAR
GTGWQGLGAYVNLGAYYLVGIPVALLLGFLVHLRTKGLWIGLLAG
ALVQGVLLAIITSLTDWKKQGGLAVKAPENKEQQMVAKSTWIEFV
EETKKIILIF
IPMLLVSTSQYLLRFVSTLMVGHVGKLYLSGAVVSMSFTNVTGFS
LLFHMGSNGAALAIGLSYWFNAIFLGLYAMYSPKCAETRAPFSME
VFSTIKDFFQ
IGIPSALMVCLEWWAYEIVILLAGVMKNPQLETSVLSISITVAIL
HSFASNSLSVAVSVRVSNELGAGNAKAIQRTVWAVLFLGFIEVGI
SAAVLFSLRY
VLGRAFVSDNQIVDYVRRMIPLICLTMILDNIHGILSGVARGTGW
QRLGAYVNLGSYYLVGIPVALILGFLVHLRAMGLWIGLVTGGLVQ
SVLLAIITSL
TDWKKEVEDTRVRVLETNLSFLQFKLKGKFRVLARKSSCNLNGLG
LRLLGLAKAKEEAMADKKGGVAVKEPENKEKQMVATSTWKEFVEE
TKNIALIFLP
MLLVSTSQYLLRFMSTLMVGHVGTLYLVVSMSFTNVTGFSLLSQY
LITHLLVISVATLAFHVTVCWDFVFKFHMGSDGAALAIGVSYWFN
AVILGLYAMY
SPKCAETRAPFSMEVFSTIKEFFQIGILSALVICLEWWAYEIVIL
LAGVMKDPQLETSVLSISNLNYSNSITVAILHSFASNSLSAASSD
STFHPAICFG
TCICKQYPNSGVDYVRRMTPLICLTMILDNIHGILSGVARGTAWQ
RPGAYINLGSYYLVGIPMAVVLGFVLHLRAKGLSIGLVIGGRRYE

GKGA
>CsMATE2
MGMSCAMETFCGQSYGAKQYHMLGIHMQRAMFILLLVSIPVAVIW
ANTGIILKVLGQDLAISEEAGLYAHYMIPSLFAYALLQCQIRFLQ
TQNIVFPMLV
SSGITTLLHIFVCWILVYKSGLGSRGAALATSISYWIHVVLLALY
VKFSSSCAKSWTGFSKEYLRDIPTFIRLAVPSVNSLELWSFEMMV
LLAGLLPNPQ
LETSVLSISLTTAENCWMIPFGLGASVSTRVSNELGASHPQTARL
AVYVVFVMAITVGILVGVVLLLIRYIWGYAYSNEVEVVRYVATMM
PILAASNIFD
GIQCVLSGVVRGCGFQKIGAYINLGSYYLVGLPCAVLFAFVLHFG
GQGLWLGIICALLVQVFLLLIVTARIDWNEEAKKAKDRVYDLAIP
LISVMFVGHL
GELALSGASMAISFANVTGFILLMGMSCAMETFSGQSYGAKQYHM
LGIHMHRAMLILFLVSTSVAVIWANTGIILKVLGQDLAISEEAGL
YAQSGLGSRG
AALANSTSYWINVVLLALYVKFSSLSAKSWTGFSKESLQAIPTFI
RLAVPSDYRFLQVLWSSKILNLCNPPSPPLEIVVIRNDGSGLWSS
SNPQLETSVL
SISLSTAEICWMIPYGIGASVSTRVSNELGAGHPQTARLVGYAYS
NEVEVVRYVATMMPILATSNIFDGIQCVLSGVVRGCGFQKIGVYI
NLGSYYLVGI
PWAVVFAFVLHIGGQAKKAKDQVYDSAIPVEIFP
>CsMATE3
MADEEGCVAVKTPESEEKQIVAQLTWNELVAEAKKIGFIFLPMLL
VTTSQYLLTFVSTLMVGHVGKLYLSGAVVSMSFTNVTGFSVLCGM
ASALETLCGQ
AYGAKQYKKLSTYTYGAIISLLLLCIPVAISWIFMEKLLIIIHQD
PLISHEAGRFSVWSIPALFSCAILQPLVRYLQSQYLVLPLLASSV
ATLAFHIPVC
WALVFKFHMGSDGAALAIGLSYWFNAIILGLYAMYSPKCADTRAP
FSVEVFSTIKKFFQIGIPSALMVCLEWWAYEIVIFLAGVMKNPQL
ETSVFSISMT
VSVLHSFAPISLAVAASVRVSNELGAGNAKAVRRTVWVVLVLGII
EVSISAAVLFSLRYDLGRAFVSDNQIVDYVRIMTPFICLTMFLDS

IHGILSGVAR
GTGWQGLGAYVNLGSYYLVGIPVAILLGFLVHLRTKGSLDWFSNR
RSGAQCSSCNYNKFNRLEKRGRRHEGKGAGKESFSGSLRRSVAIR
LPARLHFKDM
TSYRSSDTVMFLPSSLLHDYHYGMATALETLCGQAYGAKQHKKLS
IYTYGAMISLLLLCIPVAILWIFTEKLLILIHENPLISHEAAKFS
IWSILALFPY
AILHPLIRYLQLQDLIIPLLVSSVATLAFHVPVCWALVFKFIWEV
MEQHWLLVYHISSMLSFLAFMPCIHRTVLRLVFHFLMEVFSTIKE
FFQIGIPSAL
MIW
>CsMATE4
MDKGEQISSSSLNKPLIQSNGDQHSGQIKNEKGVITREDIVEEVK
KQLWLAGPLICVSLLQSCLQLISVMFVGHLGELALSGASMATSFA
SVTGFSLLMG
MSCAMETFCGQSYGAKQYHMLGIHMQRAMFVLSLVSIPLAFVWVN
TGIILKALGQDPAISEEAGRYAQYMIPSLFAYALLQCHVRFLQTQ
NIVFPMMVSS
GITTLLHVFLCWILVFKSGLGSRGAALANSISYWINVVLLALFVK
FSSSCAKTWTGFSKESLQNIPTFIRLAVPSAVMVCLEMWSFEMLV
LLSGLLPNPQ
LETSVLSVSLNTAANCWMIPFGLSASISTRVSNELGAGHPQTARL
AVYVVFVMAITEGVLVGVVLLLIRNIWGYAYSNEVEVVRYVAIMM
PILATSNFFD
GIQCVLSGVVRGCGFQKFGAYINLGAYYLVGLPCAVLFAFVLHIG
GQGLWWGILCALLVQVFFLLIVTARINWNEEAKKAEHRVHDSAIP
VELIL
>CsMATE5
MEEIDSITKLPLLSPRAGLDGDQTPRFLLGRSSVYSFASYASLFV
PDADDIDPIKGCRDFFREFMAESRKLWCLAGPAIFTSICQYSLGA
ITQTFAGHLG
SLELAAFSVENSIIANFCFGILLGMGSALETLCGQAFGAGQIDML
GVYMQRSWIILNCSSLMLMLLYIFAAPLLRLIGQTEDISREAGKL
AVWMIPQLFA
YAMNFPIAKFLQSQSKIMAMAWISAAVFGLHTLFSWLFMLKLGWG
LAGGAVVLNLSWWLIVLAQLVYILCGTCGEAWSGFSWKAFKNLWG

FVKLSLASAV
MLCLEIWYYMLLVLFAGYLKNAEIAVDALSICTNIVGWAVMVSVG
CNAAISVRVSNELGAAHPRTAKFSVVVVVLTSFVIGLFFSLVLII
FRRQYPSLFT
NSEEIKKVVNGLTPLLATCLLINNIQPALSGVAIGAGWQAVVAYV
NIGCYYVCGVPLGLILGYKLDMGVTGIWIGMLGGTVIQTLVLMWM
AYKTNWNKEA
SIAEKRIKQWGGEQQDHAAGADQEATV
>CsMATE6
MENTSDRQPLLSNENDQTLASNFLRQANHTIESSSFVADAADIPK
ITRFGEFYTQFIVESKKLWYLAGPAIFTSLCQYSLGAITQTFAGQ
VGTLDLAAFS
VENSVIAGFSFGIMLGMGSALETLCGQAYGAGQVNMLGVYMQRSW
VILNSTAFLLMFLYIFAAQLLRLIGQTEDISREAGKVAIWMIPQL
YAYAIYFPIS
KFLQSQSKIMVMAYIAGVALVLHSVLSWFFMMKLGWGLAAGTAVL
DFSWWFIVVAQMMYIFMGYCGEAWSGFSWRAFDNLWGFVKLSVAS
AVMLCLETWY
FMALILFAGYLKNAEIAVDALSVCMNLLGWTVMAAIGFNSAISVR
VSNELGARHPRTAKFSVVVVVISSFLLGLFISIILIIFRQQYPAV
FSSSEEVQKV
VYTLTPLLATCIVIDNIQPALSGVAIGAGWQAIVAYVNIGCYYVF
GIPLGLTLGYILNMGVTGIWIGMLTGTVVQTLVLFLIVYRTNWNK
ESSIAGERIR
EWGGETKDNKIKDLEN
>CsMATE7
MDEEAGQGVHENLLIPHSRKNDYELGVKKRWYNISDSAISELKQQ
TRLAGPLVLVSLLQYSLQTISVMFVGHLGEVYLSGASMATSFAGV
TGFSLMLGMG
SALETFCGQAYGAKEYHMLGVHMQRAMLVLMLICIPISILWSFTS
DIFTFLGQDPEISVQSGIYACCLIPAIFSYGLLQCQFRFLQTQNN
IKPLVISTGI
TSLVHVFICWTLVFHFGFGSRGAAISSGISYWVNVLILGIYIKFS
PTCEKTWTGYSMEGVKNLSTFLSLGIPSSLMVCLEFWSYEFLVLM
SGLLPNPKLE
TSMMSICLNTCSVFFRIPYGFGSAVSTRVSNELGAGNPQAAKQAV

QVVLFMVVVEGMLVASALVAVRGVWGYIYTNEEEVVKYIATVLPV
LAVSNFMDGM
QGVLSGATRGCAMQKVGVYVNLGTYYIIGLPLAIFLTFVLHQNGK
GLWTGIIGGSSVQAVILLLIILRIDWEQQAKKAMDLVYGLKIPGE
NVHCSGYQHD
>CsMATE8
MEDTVNEKLLTEVRRAEDGVDGEATLKDKIWVESKKMWIVAGPAI
FTRFSTFGVSIISQAFIGHIGPTELAAYALVSTVLLRFANGILLG
MASALETLCG
QSYGAKQYDMLGVYLQRSWLILFLCSIVLSPVFIFTTPILIALGQ
DESIAEVAGTISLWLIPVIFSFIASFTCQMFLQAQSKNMIIAYLA
AFSLAIHVFL
SWLLTVKYKFGLSGAMVSTILAYWIPNIGQLVYIFAGWCPETWKG
FSVLAFKDLSPIIKLSLSSGVMLCLELWYSTILVLLTGNMKNAEV
AIDALSICLN
INGWEMMISLGFLAAASVRVSNELGRGSSRAAKFSIVQIVLTSFA
IGFVLFIFFLFFRGRLAYIFTESTEVAAAVADLSPLLACSILLNS
IQPVLSGVAV
GAGWQSTVAYVNITCYYLIGIPIGVVLGYVLQLQVKGVWVGMLFG
TLTQTVVLVIITYKTDWEKQVSVAKQRVSKWSVGSDQGDEQNA
>CsMATE9
MEGSVNEELLRNVETAEGVDEQVKLKDRIWMETKKMWRVAGPAIF
TRFSTAGVAVISLAFIGHIGPTELAAYALVSTVFRRFANGVLLGM
ASALETLCGQ
SYGANQYDMLGVYLQRSWLILFLSSVALLPLFIFTTPILIALGQD
ESIAKVGGTVSLWLIPVIFSYAVSYTCQMFLQAQSKNKIISYLAL
LSLVIHVFLS
WLLTVKYKFGLSGAMISTNLAFWIPDIGQLVYICGGWCPDTWKGF
SMLAFKDLWPIIKLSLSSGVMVCVELWYNTILILLTGYMKNAEIA
IDALSICLNI
NGWEMMIALGFLAAASVRVSNELGRGSSKAAKFAIMQIVLTSFAI
GFLLFLFFLFLRGRLAYIFTESIDVVAAVADLSPLLACSILLNSI
QPVLSGVAVG
AGWQSTVAYVNIACYYLIGIPVGVVLGYVLDLQVKGVWIGMLFGT
LIQTIVLMIITYKTDWEKQVSVAKQRVKKWSVDSSQQPNADEQNA
>CsMATE10

MEGEKGNNLTQRLLGEETRSAGEVGLKWRNVELWKKESKKLWHIV
GPSLFTRVTSYTMNIVTQALAGHLGDLELASISIGNTVVVGFNFG
LILGMASALE
TLCGQAFGAKKHHMLGVYLQRSWIVLFFCCFLLLPIYIFTAPLLK
LLGQPDDVAEQTGIVALWFIPMHFSFAFLLPMQRFLQSQIKIGVM
AWVSLAVFLI
HGVVSWIFVYQFQFGVIGIALTLGMANWLIFLWLLWYVLQGGCPE
TWTGFSMEAFSGLWDFFKLSAASGVMLCLENWYFRILILMTGYLK
DATVAVDALS
ICMNVNGWELMIPFAFFAATGVRVANELGAGNGKAAKFATVVSVC
HSSMIGIFFSMVLLVLHDKYALIFTTSSQVLEAVDKLSYLLAITI
LLNSIQPVLS
GVAVGSGWQSKVAYVNLGCYYLFGIPLGIVFGWTFNFGVQGMWGG
MIIGGTAMQTIVLAIMTLQCNWEKEAENAVMHVQKWSSVRANPTD
EQS
>CsMATE11
MSGNSFHKASNANRMELEFKAQDGTTLASSSELELPHLIGANVST
EEEEDVCPTCFEDYTAENQKVITKCNHHFHLSCILEWMERSNTCP
MCNQRTGKGC
RYEGILIIGSRDYWPTSSMCFSFKDLQIAVYMQNVLGDCGFTGRR
DGVGSYHILSTRLSIVVPFSLLQTEAVKAMYSSICHLKVVPYDNE
LFFVGGATSM
EVELHHRPSEVLVTDAGRDYPAAHGFDDVINICYLESKKSWAIAA
PIAFNILCNYGINSFTTIFVGHIGDVELSSVAISLSVIANFSFGF
LFGMSSALET
ICGQAYGAGQIEMLGIYMQRSWLIMLVACICISPFYIYSTPLLKL
LGQREDIAELAGKFSIQIIPQMFSLAINFTTQKFLQAQSDVSILA
WVAFVALLFH
IATLYMFIEVFNWGTAGAAAAYDISGWVISAAQVIYIVSWCKDSW
TGFSWLALEELWEFSKLSVASAVMLCLEMWYFMTIIVLTGHLEDP
IIAVGSLSIC
MNVNGWEGMLFVGVNAAISVRVANELGSGHARAAKYSVIVMVTQS
LLIGILFMSLIMTTRNHFAVIFTDSAELQKAVAKLAYLLAITMVL
NSVQTVISGV
AVGGGWQALVAYINLGCYYIFGLPLGFLLGYKAHLGVEGIWMGMI
SGTSLQTVVLSIIILKTNWDTEVEEASERMRKWGAVE

>CsMATE12
MCQLTSSPRCCECKIDQSSYLIIPDCKNPEPDMFTRLIPNTPTTN
TKQHQTHLSLAIKEAFSIANIALPMILTGLLLYSRSMISMLFLGH
LGELALAGGS
LAVGFANITGYSILSGLAMGMEPICGQAFGAKKYTLLGLSLQRTI
LLLLVTSFPVAILWLNMRKILLFCGQDEAIAMEAQSYLMYSLPDL
LAQSLLHPLR
IYLRTQSITLPLTFCATLSIILHIPVNYLLVIKLGLGTKGVALSG
VWTNFNLVASLIIYILISGIYKKTWDGLSTECLKGWKSLLNLAIP
SCISVCLEWW
WYEIMILLCGLLVNPRATVASVGILIQTTSLIYIFPSSLSFSVST
RVGNELGANRPAKAKLAAIVGLSCSFVLGFSALFFAVSVRNVWAT
MFTQDKDIIA
LTSLVLPIIGLCELGNCPQTTGCGVLRGTARPKIGANINLGCFYL
VGMPVAVGLGFYLKLDFQGLWLGLLAAQASCAVTMLIVIARTNWE
VQAERAKELT
AGTAVVVDQIVEEEEEEKPLKAENKDYSLC
>CsMATE13
MEEPLLEAAANHQKSKGAVSVLVEVEASSWSISRWEMVMGEIKKV
SYIAMPMVVTTVSQNLLRVISMMMIGHLGELSLSGAAIATSLTNV
TGFSLLLGMA
SALETLCGQAYGAKQYQMIGSYTYGAIISLLLVCIPISILWIFTE
KLLVLIGQDPLISHEAGIYSIWLIPALFPYAVLQLLIRFLLTQSL
IYPMLLSSVA
ALVFHIPISWLLIFKFKFGSAGGALGISLSYWLNVILLGTYVKYA
SSCEKTRISFSKDVFPSIREFFRFGIPSAVMICLEWWSYELVILL
SGLLTNPQLE
TSVLSICLVVTSLHYFIPYSFGAGASTRVSNELGAGNPEAARLAA
WVATFLAAIEVIIASTILFSCKSILGYAFGEEKEVVDYVKEMTPL
LSLSIIVDCL
AALFCGVARGVGWQRIGAYVNLGAYYLCGIPMAIFLAFVLNWRGK
GLWIGLTTGSLLQSCMLMMITFFTDWKQQAREARQRIFERRSQLT
IE
>CsMATE14
MEHTVNKRLLRQVRTAGVVDEDSPLIGRIWTESKKMWIVAAPAIF
TRFSTLGVNVISQAFIGHIGSTELAAFALVSTVLLRFAIGILLGM

ASALETLCGQ
SYGAKQYDMLGIYLQRSWLIWFLCSIVLSPVFIFTTPILIALGQD
ESIAKVAGIISLWLIPVIFSFIVSFTCQMFLQAQSKNMMIAYLSA
FSLAIHVLLS
WLLTVKYDFGLSGAMISTILAFWIPNIGQLVYVFGGWCPETWRGF
SMLAFKDLFPIIKLSLSSGVMLCLELWYNTILVLLTGNMKNAKVA
ISALSICLNI
NGWEIMIALGFFAAASVRVSNELGKGSSRAAKFSTVLIVLTSFAI
GFVLFTFFLFFRGRVAYIFTKSTEVAAAVADLSPLLACSLLLSSI
QPVLSGVAVG
AGWQSTVAYVTITCYYLVGIPVGAVLGYFIQLEVKGVWVGMLVGT
LAQTTVLMIITYKTDWEKQVSVTKQRVNKWSAESDQQAEGVEQNA
>CsMATE15
METPLLNQGAPAKLQYELIGADGDYMPARTLRHWRSVFRIESQKL
WRIAIPIAMTTLCQFGLNSLTNIFVGHLGDLELSSFSIAMGVINM
FSFGFMLGMG
SATETLCGQAFGAGQVHMLGIYMQRSWIVLGTTCIFIMPIYIFAT
PILKLTGQEDDIADLAGEVAILIIPQLFSLAINFPTQKYLQAQSK
VNVLALIGFG
NLIIHCGLLWLFIFVFKWGTTGAAIAFDITSWTAALAQVIYIIIW
CKDGWTGLSPAAFRDIWPFVKLSFSSAVMLCLEVWYMMSINLLTG
NLDNAVIAVG
SLTICSNINGWEGMVFMAISAAISVRVSNELGLGRPRATKYSVYI
TVFQSLLIGLLCMIIILIMKNHIATIFTNGKEMQEAVSKLAYLLG
VTMILNSVQP
VISGVAVGGGWQALVAYINLGSYYAFGLPLGYLLGYKANLGVQGL
WGGMIIGMILQTVLLLVVLYKTNWIKEVSVLFLLQQDINNFNILW
IAMNQNSGGG
DFLAYGALGRAENEK
>CsMATE16
MSSNGRVEEAKVPLLDYASTKETVQARHQNEDEIKIQDKVWIESK
KLWRVAGPAIFSRLANFSMFVITQAFAGHLGDLELAAISISTNVI
IGFDFGLMLG
LASALETLCGQAYGAKDYRMLGIYLQRSIIVLSVVSLLLLPVFFL
ASPLLKLLGQPDDVAELCGVVSVCLIPLHFSFVFQFPLQRFLQSQ
LKNIVIAWVS

LGALILHILLTWLIVYKLQLGVIGTAFTMNISWGITPVVLFIYIS
CGGCPLSWNGFSVEAFAGLWEFLKLSASSGVMLCLENWYYKILIV
MTGNLKNAKI
AVDALSICMSINGWESMIPLAFFAATGVRVSNELGAGNGKGAKFA
TIVAVSTSTMIGLMFWLLIMIFHNNLALIFSSSTEVLEAVNKLSI
LLAFTILLNS
VQPILSGVAVGSGWQSYVAYINLGCYYLIGLPLGIAMGWIFDQGV
MGIWAGMIFGGTFIQTLILALITIRCDWEKELLTNLQPPYQGAIV
IATTYSAFYC
NMLMQLLPNPESEQPDFESGLSACRLIYITDSSKCG
>CsMATE17
MEITETGTYKLISSIDQTTTILTLRSSEKLDAESSSELERILSEG
DAPLFERFVSATWIELRLLFHLAAPAIICYMINYVMSMSTQVFAG
HIGNLELAAA
SLGNNGIQTFAYGLLVRYLFLDTSLSYRMIGSNISTSIGMGSAVE
TLCGQAYGAHKYEMLGIYLQRSFILLTLTSVLLTVIYVFSKPFLI
LLGESEEIAS
AAAIFIFGLIPQIFAYAINFPIQKFLQAQSIVQPSAYISAATLVL
HLILTYITIYKFGFGLLGSSLVLSFSWWVVVVGQIIYILKSPLCK
HTWTGFSTKA
FRGLWSFFKLSAASAVMLCLEVWYFQILVLLAGLLKRPELALDSL
AICTTINGWVFTISCGFNAAASVRVGNELGAGHPKTAAFSVVIVN
MVSFLINVIA
AIVVLLLRDSISYAFTDGEHVANAVSDLCPLLATGLVLNGIQPVL
SGVAVGCGWQAFVAYVNVGCYYAVGIPLGVLLGFYFDMGAKGIWS
GMIGGTTMQT
LILIWVTYQTDWKKEVDLATKRLDKWDNRNNKEPLLLKD
>CsMATE18
MLTNPLMVNEEGTILKQDLIKKNSTSHASLAFKELKCMANISLPM
MLTGLILYSRSMISMIFLGRLGELSLAGGALAIGFANITGYSILS
GLAMGMEPIC
GQAFGAKRFKLLGLVMQRTILLLLLISIPIAFLWCNMKKILIFCG
QEDDIATQAQLYLLFSLPDLFAQSLLHPLRIYLRSQSITLPLTFC
ATLSIILHIP
INYFLVVVLDLGIKGVALSGVWTNFNLVGSLIVYIIVSGVYKKTW
GGISTECIKGWKSLLNLAIPSCISVCLEWWWYEIMILLCGLLVNP

RATVASMGIL
IQTTSLIYIFPSSLSFGVSTRVGNELGANQPNKAKLATIIGLASS
FTVGFSALFFAIMVRNVWAKMFTLDADIIALTSMVLPIIGLCELG
NCPQTTGCGV
LRGTARPKLGANINLGCFYLVGMPVALWLGFYAGLDFRGLWLGLL
AAQGSCVITMFIVISRTNWEHQAQRAQELTGSTNVNDVGEDEKVV
DDQSKVNLKH
NDNVNTNV
>CsMATE19
MEGGVNENLLTEVRRAEEEGNLKDRVWTEYKKMWVVAGPAIFSRF
STLGVSIISQAFIGHIGPTELAAYALVSIVLLRFANGIQLGMSSA
LQTLCGQSYG
AKRYDMLGVYLQKSWLILFICSIVVTPVFIFTSPILIALGQEESI
AEVAGTISLWLIPGLFSFMVSYTCQMFLQAQSKNIIIAYLAAFSL
AIHVFLSWLL
TVKYKLGLSGAMCSTILAFWIPNIGQLIYLFGGWCPETWKGFSML
VFKDLWPIIKLSLSSGVMVCLDLWYNTILVLLAGSMKKADISIDA
LSICLNINYW
EMMISLGFMAAASVRVSNELGRGSSRAAKFSIVQILLTAFAIGFA
LFMFFLFFRGPLTYIFTESTEVAAAVADLSPLLACSILLNSIQPV
LSGVAVGAGW
QITVAYVNITCCYLIGIPIGVVLGYVLQLQVKGVWVGMLLGTLSQ
TTVLVIITYKTDWEKQVSIAEQRINKWSVDSDQESDADELNA
>CsMATE20
MEDTVNERLLREAPGAEEVVDGKKPLKDRIWMESKKMWIVAGPAI
FTRFSTMGVSVISQAFIGHVGSTELAAFALVSTVLLRFAIGILLG
LASGLETLCG
QSSGAKQYDMLGVYLQRSWLILFICSIVLSPIFIFTTPILIALGQ
DESIAQVAGTIALWLIPVIFSFIVSFSCQMFLQAQSKNLIIAYLA
AFSLAIHVFL
SWLLTVKYNFGLSGAMVSTILAFWLPNIGQLVYIFGGWCPETWKG
FSMLVFKDLLPIVKLSLSSAVMIGVKSEACTQTTALSANMIFLEL
WYNTILVLLT
GNLKNAEVAIDALSICLNINGWELMISVGFLAAASVRVSNELGRG
SSRAAIFSIVQTVLTSLAIGFVLFIFFLFFRGRLAYIFTESTEVA
AAVADLSPLL

AFSILLNSIQPVLSGVAVGAGWQSTVAYVNITCYYLIGIPVGIVL
GYVLQFQVKGVWVGMLIGTLTQTIVLMIVTYMTDWEKQVSLAKQR
INKWSVESDQ
QADDEQNA
>CsMATE21
MLANNLVIDHHHHPEGLILKQEEIIKNNSTVTTTTDHFSIFSQES
KCIANIALPMILTSLLLYARSMISMLFLGRLGNLSLAGGALAIGF
ANITGYSILS
GLAMGMEPICGQAFGARRYKLLGLTMQKTILLLFLTSIPLCFLWC
NMKKILLFCGQDDEISTQAQLYILYSLPDLFTQSLMHPLRIYLRS
QSITLPLTYC
AILAILLHIPINFFLVSILNLGIRGVALAGVWTNFNILGSLIVYI
LVSRIHEKTWGGISLECLNDWKALLNLAIPSCISICLEWWWYEIM
ILLCGLLTNP
RATVTSMGILIQTTSLIYIFPASLSFGVSTRVGNELGANQPEKAK
LAAIIGIFWSFILGFSALIFSISVRKIWACMFTKDTEIIALTSMV
LPIIGLCELG
NCPQTTGCGILRGTACPELGARINLGCFYLVGMPTAVWLGFFIGL
DFVGLWLGLLAAQGSCVVTMLLILSRTNWDHQAQRSKELTGTIED
IKEEDKNVVD
DETNILVEDKLEKLNLV
>CsMATE22
MQTISPRSLEYEIFTENLLSNLKSYGTSLVLLFLPSICQYSLGAI
TQTFSGHVGTLQLAAFSVENSVIAGLSFGVMMGMGSALETLCGQA
FGAGQIDMLG
VYMQRSWVILNCTSLILMFMYIFAAQFLRLIGQTEDISREAGKLA
IWMIPQLFAYSMNFPIAKFLQAQSKIMAMAWISAAALVVHTFFSW
LFMLKLGWGL
AGGAVVLNFSWWFIVLAQLAYIFSGTCGEAWSGFSWKAFQNLWGF
VRLSLASAVMLCLEIWYFMALVLFAGYLKNAEIAVDALSICTNIV
GWAVMVAIGC
NAAISLGLRVGCGCVRVSNELGAAHPRTAKFSVVVVVLSSFVIGL
LLSLILIIFRKQYPSLFTNSEEVKRVVYGLTPLLATCLVINNIQP
ALSGVAIGAG
WQAIVAYVNIMCYYIFGIPIGLLMGYKLDMGVKGIWIGMLSGTVV
QTLVLFWIVYRTNWNKEASIAEQRIRQWGGERDQRAKGLEN

>CsMATE23
MEEPLLATADNCRKDQGASSVVVLVEPETLLSSSSLSLLRWEVII
SEIKKVVYIAMPMMVTTVSQYLVRVISMMMIGHLGELSLSGAAIA
TSLTNITGFS
LLLGMASALETLCGQAYGAKQYQLIGSYTYGAIISLLLVCIPVLI
LWCFTEKLLVLIGQDPLISHEAGIYSICLIPALFPYAILQLLVRY
LLTQSLLYPM
LLSSVAALVFHIPLCWVLIFKFKFGSDGAALAISLSYWLNVIILG
IYVKYASTCENTRISFSKDVYPSIPEFFRYGIPSAVMICLQWWSG
ELAILLSGLL
LNPQLETSVLTICITVASLHYYISYSFSTGASTRVSNELGAGHAE
AARLAAWVATFLAVVEVVIASTILFSCRSIFGYAFGDGKELVDYV
KEMTPFLCFT
IMTDCLANMFSGVARGVGWQRLAAYVNLGAFYLCGIPIACVLAFV
YHWRGKGLWIGIATASFLQSGMLMMITIFTDWKKQARNTRKRIFE
GRSQLSTIE
>CsMATE24
MGDAEAAVEVKSPENKEELMVAKSTFNEFVAEAKKISFIFMPMLI
VTASDYSLRFLSTLMVGHVGKLYFSGAVISTSITNVIGFSFLYGM
AGALETLCGQ
AYGAKQHKKLSIYTYGAIITLLLLCIPIAIIWLFMEKLLILIQQD
PQISRVAGEFSIWLIPALFSHAILQPLIRYFQSQYLIFPLLATSV
ATLAFHIPMC
WAFVFKFNMGSNGAALAISLSYWLNTIILGLYAMYSPKCAETRAP
FSMEVFSTIKDFFRLGIPSALMACLEWWAYEIIILLAGIMRNPEL
ETSVLVTVAI
LHYLTPYSLGVAASVRISNELGAGNPKAARRTVWVVMVLGVTEVS
ISAAVLFSLRHVLGRAFVSDKQIVDYVRRMTPFICLTMILDNFQA
ILSGVARGTG
WQTLGACVNLGSYYVVGTPVAILLGFQAHLKAKGLWIGIVAGALV
QSILLAIITCFTDWKKEVENTRERVLDLNVNTDERGKVLPLGSLQ
AEEMTSFE
>CsMATE25
MEEPLLQTSNNHGVASVIVSEMKKVSYIAMPMAVTTVSQNMIRVI
SMMMIGHLGELSLSGAAIATSLTNVTGFSLLFGMASALETLCGQA
YGARQYHMIG

TYTYGAIISLLLVCIPISILWIFTEKFLLMIGQDPSISHEAGIFS
IWLIPSLFPYAILQLLVRFLLTQSLLYPMLLSSVAALVFHIPLSW
VIIYKFKFGS
GGAALAIGLSYWLNVILLGIYVKFTPLCEKTRISFSKDVFPSIRE
FLRFGIPSAVMICLEWWSYELVVLLAGLLANPQLETSVLSICLTI
AALHYYIPYS
FGAGASTRVSNELGAGHPEAAKLAAWVATFLAAIEVAIASTILFS
CRSILGYAFGGEKEVVDYVREITPLLRVARGTGWQHLGAYVNLAA
YYAGGIPIAC
MLAFVFHWGGKGLWIGLTIGSFLQTFMLMMITFFTDWKKQAREAR
ERIFEGRSQLTIE
>CsMATE26
MEEPLLKTTDRGLENQGGASVVVVEEETLSLSSSLPRRWDVIACE
MKKVSYIAMPMVVTTVSQNLLRVISMMMIGHLGELSLSGASIATS
LTNVTGFSLL
FGMSSALETLCGQAYGAEQYQMIGTYTYGAIISLLLVCIPISILW
IFTEKLLILIGQDPLISHEAGIYSIWLVPTLFPYAILQLLTRFLL
TQSLIYPMLL
SSVAALVFHIPISWLLIFKFKFGSAGAALGIGLSYWLNVILLGIY
VKYASSCEKSRISFSKDVFPSIREFFQFGIPSAVMICLEWWSYEV
AILLSGLLPN
PQLETSVLSICLVVSSLHYFIPYSFSAGASTRVSNELGAGNPEAA
RLAAWVATFLAVIEVVIASAILFSCRSILGYAFGEEKEVIDYVEE
MIPLLRVARG
VGWQRLGAYVNLGSYYLCGIPVACVLAFVLHWRGKGLWIGLTTAS
LLQGVMFMTITVLTDWKKQVRKARERMYERKSQMTIE
>CsMATE27
MVMPMRDVEAELSEKDDITNSRKTLLKYGEELKKTSRIAAPMVAV
SVLQYLLQVISVMMVGHVNQLTLASVAIATSLTNVTGFSLLSGLV
GGLETLCGQA
YGARQYHKVGVYTCSAIISLLLVCLPVCVFWTFMDKFLILIGQDH
SISIGARKYALWVIPALFGSAILKPVVRYLQSQTLTRPLLASSFV
VLCFHVPVCW
ALIFKFKLGTVGAALAFSIEAFLVIGQFFRFAVPSAVMVCLKWWS
LEVLVLLSGLLSNPKLETSVLTICLTISTLHFTVPYGIGAAASTR
VSNELGAGNP

QAARVAVWTVIFLAAVEAVIVSTTLFFCRNILGHAYSNDKAVVSY
IAVMTPLICLSVITDSIQAVISGIARGSGWQHIGAYVNIGAFYLF
GIPIAVVLGF
TKHMNAKGLWIGIVVGSIIQSSVLFLVTSFTNWQKQATNAKERMS
KVGSTIENEEESTRNDHQ
>CsMATE28
MEDGLLIKRNDDDENNIEMKGGFRMITGVVLWEEFKKLGYIAGPM
VAVTLSVYLLQVVSVMMVGHLGELALSSTSIAFSLAGVTGFSVMQ
GMASALETLC
GQAYGAGQYQKLGIQTYTAILCLVIVCVPLSVLWVNMGYILKFIG
QDPLISREAGKFIIWLIPSLFAYATLQPLVRYFQMQSYILPLLIS
TGITFCVHVP
LCWVLVFKSGLQNVGAALAIGIKEFFLFAIPSAFMLCLEWWSFEL
LVLLSGILPNPQLETSVLAICLNTIATLYAIPYGLGAGVSTRVSN
ELGAGNPQGA
RVAVFVVVIMALSEAIIISGTIFACRNVFGYTFSNEKEVIVYVTD
MAPLICLSIIMDSLQGVFSGVARGCGWQHLGAYVNLAAFYLFGIP
IAAALGFWAQ
LRGKGLWIGIQAGAALQTILLLIITVCTNWEMQGKPNSSRAVCTD
RVNTRSCWDKP
>CsMATE29
MDDEAGQGLLQNVPHYRNDDDIELGVKKRWYIISDSTMVELTEQV
CLAGPLVLVSLLQYSLQTISVMFVGHLGEVCLSGASMATSFAGVT
GFSLMLGMGC
ALETFCGQAYGAKEYHMLGVHMQRAMLVLMLICIPISILWSFTSN
IFRFFGQDLDISVQSGIYACWLIPAVFAYGLLQCQFRFLQTQNNI
RPLVISTGIT
SLMHLLICWTLVFRFGFGSRGAAISSGISYWVNVLILGIYIKFSP
TCEKTWTGCSMEGVKNLSGFLSLGIPSSLMLCLEFWSYEFLVLMS
GLLPNPKLET
SMMSICLNTCSVFFRIPYGFGSAVSTRVSNELGAGNPWAAKLAVQ
EVVRYIARVLPVLAISNFMDGMQGVLSGATRGCAMQKVGVYVNLG
AYYIIGLPLA
ILLTFVLHQNGKGLWTGIIGGSSLQAVILLVIILRLDWEQQAKKA
INLVHGSKIPGDYVYFSKPEIAAS
>CsMATE30

MEEPLLATADNCQKDQGGSSSSSSSSLLRREVVVGEIKKVVYIAM
PMMVTTVSQYLLRVISMMMIGHLGELSLSGAAIATSLTNVTGFSV
LLGMASALET
LCGQAYGAKQYQLIGSYTYGAIISLLLVCIPISILWIFTEKLLVL
IGQDPLISHEAGIYSICLIPTLFPCAILQLLIRYLLIQSLLYPML
LSSVAALLFH
IPISWMLIFKFEFGSAGAALAISLSYWLNVILLGIYVKYASTCEN
TRISFSKDVYPGIREFFRYGIPSAVMICLQWWSYELAILISGLLP
NPQLETSVLT
ICFIVASLHYYVPYSFSTGASTRVSNELGAGHAEAARLAAWVATF
LAVIEVVIATTILFSCRSLLGYAFGDEKELVDYVKEMTPFLCCTI
MTDCLAIMFS
GVARGVGWQRLAAYVNLGAFYLCGIPLACVLAFVFHWRGKGLWIG
KKHKEEDICGEISAKYD
>CsMATE31
MHNGLHNRLLVPKVQQQEEIDLKSKVLQESKNIWRVALPGVISRV
GSFGTLVVTQSFIGHISPLDLAGYALVQTIIVRFVNGILLGMSSA
TETLCGQAFG
AGQYHMMGIYLQRSWIVDLITLTILLPVFIFGRLLFTLLGQEESI
SKRAGYVSMWFIPFVYSFVFSLTIQMYLQAQLKNKLIAWISVFQF
ILHVPLSWLF
VNYFSFGLPGAMSALIISSWFLVFGEFIYIFGGWCPHSWRGFSKA
AFMDIFPIVKLSLSSGLMVCLELWYYAVLVLIAGFMENAEVGISA
FSICLNIYGW
ELMICLGFLGAACVRVANELGKGNANAVRFSIKVLVATSLLIGVI
FSSVCFVFHKQLAQFFTNDKEVADTVSDLSVLLSISVFLNRVAVG
AGLQGTVAIV
NLVCYYLIGTPIGIYLGYVVGLQVKGVWIGMICGIVCQSITLCVL
AWRTNWDVQVLKASERLNRFYLKSEEETNQSSSHA
>CsMATE32
MDNGLHEGLLVSKVQKEEEIELKSKVWQESKNIWRVALPGIISRA
GSFGTIVVTQSFLGHISPLDLAGYALVQTIIVRFVNGILLGMSSA
TETLCGQAFG
AGQYHMMGIYLQRSWIVDLITLTILLPVFVFGRQLFMLLGQEESI
AKTGGYISLWFIPFAYSFVFSLTIQMYLQAQLKNKLIAWISAFQF
ILHIPLSWLL

VTYFSFGVPGAMSALIISSWFLIFGEFIYIFGGWCPHSWKGFSKA
AFLDILPVVKLSLSSGFMVCLELWYSAVLVLIAGYMDDAEVGISA
FSICLNINTW
ELMICLGFLGAACVRVANELGKGNADAVRFSIKVLIATSLLIGVF
LSSICFFFHKELAQFFTNDKEVADTVSDLSVLLGVSVFLNRVAVG
AGLQGTVAIV
NLVCYYLIGIPIGIFLGYVVGLQVKGVWIGMICGIACQTITLCVL
AWRTNWDVQVLKASERLKRFYLTSGEEKNRSSNDA
>CsMATE33
MESLDELHKPILHSTEPLPLPLQVGGTSTELEKVLSDTESPFLNR
IRIATWIELKLLFRLAAPTVLVYLINNSMSMSTRIFAGHLGNLEF
AAASLGNQGI
QLFAYGLLLGMGSAVETLCGQAYGAQRYEMLGVYLQRSTIVLTLT
ALPVTIVYIFSKQILLSLGESTTVASSAAMFVYGLIPQIFAYAVN
FPIQKFLQSQ
SIVVPSACISAATLVVHLILSYVAVYKIGMGLLGASLVLSLSWWI
IVLGQMVYILMSDRCKATWTGFRWEAFSGVWEFVKLSSGSAVMLC
LETWYMQILV
LISGLLENPELSLDALSVCSAVNGMMFMVSVGFNAAASVRVGNEL
GGGNPNYAFTGGETVAKAVSELCPLLAVTLILNGVQPVLSGVAVG
CGWQAFVAYV
NVGCYYGVGIPLGCLLGFKYKFGVKGIWSGMIGGTMMQTLILLWS
TYRTDWNKEVIFLVQTFYSVLPHLLRVPSSNTVECVCQAVEKSRK
RLDKWEGNAE
EALVCRIIAFQIRGGKSQKTLGPVEGNVGHLPRA
>CsMATE34
MEGSVSENLLTEVGRAEEEGTLKDRVWTESKKMWIVAGPAIFSRF
STFGVSIISQAFIGHIGPTELAAYALVSTVLLRFANGILLGMSSA
LGTLCGQSYG
AKRYDMLGEESIAKVAGTISLWLIPGLFSFMVSYTCQMFLQSQSK
NMIISYLAAFSLAIHVFLSWLLTVKYNFGLSGAMVSTILAYWIPN
IGQLFYIFGG
WCPETWKGFSVLVFKDLWPIIKLSLSSGVMVCLELWYNTILVLLT
GSMKKADIAIDALSICLNISGWEMMISLGFLAAASVRVSNELGRG
SSRAAKFSIV
QIVLTSFAIGFVLFIFFLFLRGRLAYIFTRSMEVAAAVADLSPLL

ACSILLNSIQPVLSGVAVGAGWQSTVAYVNITCYYLIGIPVGVVL
GYVFQLQVKG
VWIGMLFGTLAQTTVLMIITYRTDWEKQVLVARQRVNKWSVDSDH
LQADEDEQNA
>CsMATE35
MCNQNKTRTITFSDHYYFNLLSLPLSAKPHETTSPKEDEPFFQSC
VLSETKSLIRLAFPIALTALILYARSIISMIFLGHLGDTELAAGS
LAMAFANITG
YSVLSGLALGMEPLCTQAFGAQRPKLLSLTLQRTILFLMVFSIPI
TFLWLNISHVLKFLHQDPNITDLARTFLIFAIPDLVTNSIIHPIR
IYLRAQGITH
PLTLASLVGTILHLPINFLLVSHLRLGVAGVAAASAASNLVVLVT
VVCYVWALGIHVPTWAAPTRECLTGWMPLIRLAAPSCVSVCLEWW
WYEIMIVLCG
LLVDPKATVASMGVLIQTTSLIYVFPSSLSFAVSTRVGNELGANR
PDKARVSAVVSIFLAGLMGFCAMSFAILVKDVWAHMFTSDVNILH
LTSTVLPILG
LCELGNCPQTIGCGVVRGTARPSTAANVNLGAFYLVGMPVAVGLG
FWFDLGFSGLWLGMLAAQFCCAGLMLCVVGSTDWEDQAKKAQALT
CCESKETKLP
SGEGKETMICITVT
>CsMATE36
MEGSVNEELLRKVQTAESMDEEVKLKDRIWMETKKMWRVAGPAIF
TRFSTAGVTVISLAFIGHIGPTELAAYALVSTVLLRFANGILLGM
ASALETLCGQ
SYGATQYDMLGVYLQRSWLILFLCSTALLPIFIFTTPILIALGQD
ESIAKVAGTISLWLIPVIFSYAVSLYLPNVPSSTEQEYDNLILGI
SFTGNPCQLV
YLFGGWCPDTWKGFSMLAFKDLWPIIKLSLSSGVMICLELWYSTI
LILLTGNMKNAEVAIDALSICLNINGWEMMIALGFLAAASVRVSN
ELGRGSSKAA
KFSIVQIVLTSLAVGFLLFLFFLFFRGRLAYIFTESIEVAAGVAD
LSPLLACSILLNSIQPVLSGVAVGAGWQSTIAYVNIACYYLIGIP
VGIVLGYIFE
LQVKGVWIGMLFGTLIQTFVLMIITYKTDWEKQVSVTKQRINKWS
VDSSRQPNAEYADEQNA

>CsMATE37
MGDSEAAVAVKTPENKEKQMVKKSTWNDFVAEAKQISMIFTPMLI
VTTSQYLLRFVSTLMVGHVGKLYLSGAVVSMSFTNVSGFSFLFGM
ASALETLCGQ
AYGAKQHKKLSHYTYGAIISLLIICVPVAILWIFMEKLLILIQQD
PLISHEAGKFSIRLIPALFPYAILQPLVRYLQSQYLILPLLASSV
ATLAFHVPVC
WAFVFKFNMGSDGAAFAIGLSYWFNAIFLGLYAFYSPKCADTRAP
FTMEVFSTIKDFFRFGIPSALMVCITVAVLHYFAPFSLGVAASVR
VSNELGAGNP
KAVRTTVWVVMVLGVIEVSIASVVLFSLRHVLGRAFVSDNQIVDY
VRRMTPFICLTMILDSVQGILSGVARGTGWQRLGAYVNLGSYYLV
GVPVALLLGF
LVHMRAKGLWIGLVAGSLVQSILLLIITSFTNWKKEVEDTKERVL
EKKVSDAKK
>CsMATE38
MDKGLHERLIVPKVQEEEEIDLKSKVWQESKNIWRVALPGVISRV
SSFGTIVVTQSFVGHISALDLAGYALVQTITVRFVNGILLGMSSA
TETLCGQAFG
AGQYHMMGIYLQRSWIVDLITLTILLPMYLQAQLKNKLIAWISVF
QFLLHVPLSWLFVSYFSFGVSGAMSALCISSWFLVIGEFIYIFGG
WCPHSWKGFS
KAAFLDILPVVKLSLSSGLMVCLELWYNAVLVLIAGYMDNAEVAI
SAFSICLNINGWEFMICLGFLGAACVRVANELGKGNANAVRFSIK
VLMATSLLIG
VIFSSLCFIYHKELAYFFTNDKEVADTVSDLSVLLGVSVFLNSIY
PVLSGKSHFFRRVAVGAGLQGTVAIVNLVCYYLIGIPVGVFLGYV
VGLQVKGIWI
GMICGIVCQSITLCILAWRTNWDVQVQKASERLNRFYLKSGEETN
RSSNHA
>CsMATE39
MAPISGEDQHLSQKLLQEKGRLVTQSNDQTSLSWRFWTESKKLWQ
ITGPSILSRVAAANMNVVTQAFAGHLGQVELASISIANTVIVGFN
FGLLLGMASA
LETLCGQAFGAKRYRMMGLMGQPDDVAEQSGIVAAWLIPLHFSFA
FVFPVQSWLFVYKFQLGVVGIALTLDVAWWLLAAFQFAYVSWGGC

LETWAGFSME
AFSGLWEFVKLSAASGVMLCLENWYYRILILMTGNLDNATLAVDA
LSICMSINSWEMMIPFAFFAATGVRVANELGAGDGKAAKFATTVA
VLQSTIIGLI
FGILIMIFRVPLTMIFTSSPEVLHATEKLTYLLAFTILLNSVQPV
LSGVAVGSGWQSKVAYVNLGCYYLIGIPLGVILGWVFHLGVEGIW
GGMIFGGTAV
QTVILAIMTIRCNWELEAEKAANHIENWSRKGSNDEKQELRK
>CsMATE40
MMEEVLLVSVNKESEIRKWERFVEEMKKMSCIAMPMVLVTVSQNL
LRVISMMMVGHLGELSLSGAAVATSLTNVTGFSLMFGLASALETL
CGQAYGAEQF
KKLGVYTYGAIISLLVLCIPVSFLWLYMDEFLVLIGQDPLISLEA
GKYAMWLIPTLFPYAILQLLVRYLQSQSLILPMVLSSIAALILHI
PLCWALVFKF
NLGSSGAALSLGFSHWLNVVLLGIYVNSFTLLVPSAVMVCLEWWS
FELIIILLAGKLANPQLETSVLSICFTVTALHYYIPYSFGAAARLV
RKPFASLLGV
SNALGAGNPNAAELATMTVMVLGAAEVIIAVSVLYFCHDVLGYAF
GYEKEIVDYVKDMTPIICLSMIMDSLQAVVSGIARGTGWQHIGAY
VNLGAYYLVG
IPVALISGFVLHFGGKGLWLGFLGGALRSYEINMSSRGDFSDDFD
MQLIGNFLSFASRGDRVGLNMMLREGISPNVQDYDKRTALHLAAS
EGHASIVELL
VQYKADVNLEDRWQRTPLTDARLYGHRDICRILEVNGGKDTINHH
SMTVRPEEGSYEVDIDMSELNMQYSTTIKQIVVLYLRAAISSAVV
LQQAKLNVIG
VYGESEKVKWRGTWVVKTIFKREISHPVNMVLTATDNTTLRELRH
PNILQFLGSILHGEEMILITEHLSEGNLENILTKKTRLDLVTAMR
YALDIAR
>CsMATE41
MCQLTSSPRCCECKIDQSSYLIIPDCKNPEPDMFTRLIPNTPTTN
TKQHQTHLSLAIKEAFSIANIALPMILTGLLLYSRSMISMLFLGH
LGELALAGGS
LAVGFANITGYSILSGLAMGMEPICGQAFGAKKYTLLGLSLQRTI
LLLLVTSFPVAILWLNMRKILLFCGQDEAIAMEAQSYLMYSLPDL

LAQSLLHPLR
IYLRTQSITLPLTFCATLSIILHIPVNYLLVIKLGLGTKGVALSG
VWTNFNLVASLIIYILISGIYKKTWDGLSTECLKGWKSLLNLAIP
SCISVCLEWW
WRQPACKSQACCHCRPFLQLCVGIFSTFLAVSVRNVWATMFTQDK
DIIALTSLVLPIIGLCELGNCPQTTGCGVLRGTARPKIGANINLG
CFYLVGMPVA
VGLGFYLKLDFQGLWLGLLAAQASCAVTMLIVIARTNWEVQAERA
KELTAGTAVVVDQIVEEEEEKPLKAENKDYSLC
>CsMATE42
MGDAESDVAVKNPENNDELLVAKSTWSEFVAETKQISFIFMPMVL
VTTTQYLLRFVSTLMVGHVGKLYLSGAVLAMSFTNVTGFSFLFGM
SSALETLCGQ
AHGARQHKKLSLYTYGAIISLLLICIPVAILWIFMEKLLILIHQD
HLISHEAGKFSIRLIPALFPYAILQPLIRYLQSQYLILPLLVSSI
ATLAFHVPVC
WAFVFKFNMGSDGAALAIGLSYWFNAIFLGLYAMYSPKCAATRAP
FSMEVFGTIKDFLRLGIPSALMVCITVAVLHYFSPFALGVAASIR
VSNELGAGNP
KAVRMTVWVVMVLGVIEVSIAAVVLFSIRHVLGRAFVSDNQIVDY
VRRMTPFICLTMVLDSIQGILSGVARGTGWQRLGAYVNLGSYYLV
GIPMALLLGF
LLHLKAKGLWIGLVAGALVEDTRLRVLETKIPAEK
>CsMATE43
MHNGLHNRLLVPKVQQEEEIDLKSKVWQESKNIWRVALPGVISRV
GSFGTLVVTQSFIGHISPLDLAGYALAQTIIVRFVNGILLGMSSA
TETLCGQAFG
AGQYHMMGIYLQRSWIVDLITLTILLPVFIFGRLLFTLLGQEESI
SKLAGYVSLWFIPLVYSFVFSLTFQMYLQAQLKNKLIAWISVFQF
ILHVPLSWLF
VNYFSFRLPGAMSALIISSWFLIFGEFIYIFGGWCPHSWREFSKA
AFLDIFPIVKLSLSSGLMVCLELWYYAVLVLIAGFMENAEVGISA
FSICLNIYGW
ELMICLGFLGAACVRVANELGKGNANAVRFSIKVLVATSLLIGVI
FSRVAVGAGLQGTVAIVNLVCYYLIGTPIGIYLGYVVGLQVKGVW
IGMICGIVCQ

SITLCVLAWRTNWDVQVLKASERLNRFYLKSEEETNRSSTHA
>CsMATE44
MEDRRLLIKRNDDDNERDMNRGGFRIITGAVFWKEVKKLGYIAG
PMVAVTLSVYLLQIASVMMVGHLGELALSSTSIAFSLATVTGFSV
MAYGAGQYQK
LGIQTYTAILCLVIVCFPLSMLWINVGYILTSLGQDPMISREAGR
FIIWLIPSLFAYATLQPLVRYFQMQSYILPLLISTGVTFCVHLPL
CWVLVFKSGL
QNVGAAVAIGIKEFFLFAIPSAFMICLEWWSFELLVLFSGLLPNP
QLETSVLAICLNTIAALYAIPYGLGAGASTRVSNELGAGNPQGAR
VAVFVVVIMA
LSEAIIISGTIFVCGNVYGYTFSSEKEVIVYVTDMAPLICLSIIM
DSLQGVLSGVARGCGWQHLGAYVNLAAFYLVGIPIAAALGFWVQL
RGKGLWIGIQ
AGAALQTFLLFIVTVCTNWEMQVGR
>CsMATE45
MEEPLLETTDNCLENQGGAYVVVVEVEALSLSSSLPRRWEVILCE
MKKVSYIAMPMVVTTVSQELVRVISMMMIGHLGELSLSGASIATS
LTNVTGFSLL
FGMSSALETLCGQAYGAEQYQMIGTYTYGAIISLILVCIPISILW
IFTEKLLVLIGQDPLISHEAGIYSIWLVPTIFPYAILQLLISFLL
TQSLIYPMLL
SSVAALVFHIPISWLLIFKFEFGSAGAALGISLSYWLNVILLGIY
VKYASSCEKSRISFSKDVFPSIREFFRFGIPSTVMICLEWWSYEL
VILLSGLLPN
PQLETSVLSICLVVSSLHYFVPYSFSAGASTRVSNELGAGHPEAA
RLAAWVATFLAVVEVVIASAILFSCRSILGYAFGEEKEVVDYVEE
MIPLLSLSMM
VDCLAVLFSGLHLSSLYFINILILMRFRVR
>CsMATE46
MAQKWPGNFMQIAFFEMKKQRGLAIPLVAMNLVWFMKLAITTAFL
GRLGELQLAGGALGFTFANVTGFSVLSGLSNAMEPICGQAYGAKN
FKLLHKSLVM
TTFLLLLTTLPVSVLWINVDKILIFFGQERDIALTARKYLVYLLP
DLVITSFLCPLKAYLSSQSLTIPVMLSSTVAVALHVPINILLMKA
KGLAGVSMAV

WISDLIVVVLLTVYIVRLEIKKGGKWEGGGWFEQGTKDWINLLKL
CGPCCLTTCLEWWCYEILVLLAGWLPNARQAVGVLAIVLNFDYLV
YSVMLSLATS
ASIRVSNELGANQPGLAYQSACVSLAVSSITGCIGGAVMVVSRGI
WGPLFSNNKGIISSVKKMILLMALIEVVNFPLAVSGGIVRGTARP
WLGMYANLGG
FYLLALPLSWVLAFKVHLGLGGLLAGFFVGMVGCLILLLVFVARI
DWVEEAGKAQLRTSHLEEDQEAVSKDPKYNLQTPIDAVI
>CsMATE47
MEQPLLPQLAKNETLSSSSLSSLSISRREVVMREIKKVSYIALPM
VVTTVSQSLLAMINMMMIGHLGELALSGAAIATSLANVTGFSLMI
GMSGALETLC
GQAYGAKQYQMIGTYTYAAIISLILVCIPVSILWCFTEKFLIVIG
QDPLISHEAGIYIKWLIPALLPSAIIQVLVRFFLTQSLLYPMLLS
SVAALIFHIP
VTWLLMFKFNFGSAGAALGLGLACWLNVLVLGIYVKYTSSCEKTR
ISFSKNVFPCIREFFRFGIPSAFMLCLEWWSYELAILLSGRLPNP
QLETSVMSIC
FVVSSLHFNIPNSFAAGASTRVSNELGAGNPEAARLAAWIATVLA
VIEVVIASTVLFSFRSILGYAFGGEKEMVDYIKEMIPLISLSIMG
DCLATLFSGI
HLSAL
>CsMATE48
MEDPLLVTANKHLKNQGAASVGGGGGGNSCHSHYQKDGRVISMMM
VGHLGELSLSGAAIATSLTNVTGFSLLFGMASALETLCGQAYGAG
QYQMLGTYTY
GAIISLLLVCIPISFLWIFTEKLLIMIGQDPLISHEAGIYSIWLI
PTLFPFTILQAVGSLLADPKFVISNAFKFCCSSSFPHTYIMVIDI
QVQIWKWRGS
IRHWITFSKDVFPSIREFFRFGIPSVVMICLEWWSYELAILLSGL
LPNPQLETSVLSICLTVSSMHYFIPYSFGAGASTRVSNELGAGNP
QAAKLAAWVG
TFLAVLEGAVASTILFSCKSILGYAFGKEKEVVDYVEEMTPILTV
ARGTGWQHLGAYVNLGAYYLCGNPMAYVLAFVLHWRGKGLWIGLT
TASLLQGLML
MMITFFTDWKKQAREARVRIFEGRSQLTIQ

>CsMATE49
MGMSCAMETFCGQSYGAKQYHMLGIHMQRAMFVLSLVSIPLAFVW
ANTGIILKALGQDPAISEEAGRYAQYMIPSLFAYALLQCHVRFLQ
TQNIVFPMRV
SSGITTLLHVFLCWILVFKSGLGSRGAAFANSISYWINVLLLAFY
VKFSSSCAKTWTGFSKESLQNIPTFIRLAVPSAVMVCLEMWSFEM
LVLLSGLLPN
PQLETSVLSVSLNTAANCWMIPFGLGASIRSSTNCTFGGVCGLCH
GYYGGVLVGVVLLLIRNIRGYAYSNEVEVVRYVAIMMPILATSNF
FDGIQCVLSG
VVRGCGFQKFGAYRNLRAYYLVGLPCAVLFAFVLHIRGQNPAHLF
DEGPLVGDPLRIISSTRRQNMEFTILQSLLK
>CsMATE50
MAEKVETYPCQESWKIFCCPFLKDLRLVFKLDELGLEIAKIAFPA
AMALTADPIASLIDTAFIGQIGAVELAAVGISIALFNQVSRIAIF
PLVSVTTSFV
AEEDTITQLSQEAQESETLQMGSVVSTEGDKLIPIEESDDCTKML
ESHPKSCNIVKTESGKRSIPSASSALIIGGILGIIQAVLLISLAE
PALNIMGVKS
DSPMLNPAQQYLKLRSLGAPAVLLSLAMQGVFRGFKDTQTPLYAT
LAGDITNIVLDPIFIFVFHLGVSGAAIAHVLSQYLISVVLFWRLV
QQVVLIPPSR
KHLQFARFLKNGFLLLMRVISVTFCVTLAASMAAHQGPVPMAAFQ
VCLQVWLATSLLADGLAVAGQAILASAFARKDFDSTTATASRVLQ
LGLVLGLVLA
IILGVGLPFAGRIFTKDGDVLHLIRVATPFVAATQPINALAFVFD
GVNFGASDFSYAAQSMVLVAILSIISLFILSSSLGYVGLWIALTI
YMSLRAFVGF
WRIGTGTGPWSYLRN
>CsMATE51
MAEDSAPAKGKMPIMVFFRDVRLLFKMDSLGSEILRIAFPAALAL
AADPIASIIDTAFIGHLGAVELAAVGVSIAIFNQASKVTIFPLVS
ITTSFVAEED
TIGRLAKEMQMNEMVKPKAEDVILDSLEKGSSATNMETQEAKADN
STLSILATLASETSASKPQNVACCDSDKTQTKAKKHQSTDSQTNV
ARVKRHIPSA

STALVMGAVLGILQTIFLISLARPLLGIMGVRSDSPMLEPAKRYL
KLRSLGAPAVLLSLAMQGVFRGFKDTTTPLYATVAGDLANVVLDP
ILIFVCHMGV
SGAAIAHVLSQYLILVILFWKLTKQVYLLPPSLKSLQFSRFLKNG
FYLLGRVIAATSCVTLAASLAARLGTIPMAAFQICLQVWMASSLL
ADGLAVAGQA
IIACAFAERDYKKATSAASRVLQMAFVLGLGLSLVVGLGLQFGSV
VFTKDKHVLHLITLGVPFVAATQPINSLAFVFDGVNYGVSDFAYS
AYSMVLVALL
SITSLFLLSDSNGFVGIWLALTIYMSLRALAGVWRMGTGTGPWSF
LRGL
>CsMATE52
MAEDGVIHSPAAKGKMPFMVFFRDVRLLFRMDSLGSEILRIAFPA
ALALAADPVASLIDTVFIGRLGAVEIAAVGVSIAIFNQASKVTIF
PLVSITTSFV
AQEDTTERLAKEMQIKSEMVKAKADDIILDNLEKGGSVTNIETKE
VKDVADDIILDNLEEGGSVMNIETKEVKDVLTTSACETDASESQN
GDCPELNKTQ
TKAKKHQSTDSETKSPREKKHIPSASTALVVGAVLGILQTIFLSS
LAKPLLGIMGVGSDSPLLKSAKRYLRLRSLGAPAVLLSLAMQGVF
RGFKDTTTPL
YATVAGDLANIVLDPILIFVGRLGVGGAAIAHVLSQYLIVVILFC
KLLKQVHLIPPSIKSLQFNRFLKNGFYLLGRVIATTSCVTLAASL
AARLGTTPMA
AFQVCLQVWMTSSLLADALAVAGQAIIACAFAEKDYLKATSAASR
VLQMAFVLGLGLSLIVGLGLQFGSVAFSNDKHVLHLITIGVPFVA
ATQPINSIAF
VFDGVNYGVSDFAYSAYSMVVVALLSTASLFLLSKSNGFVGIWLA
LTIYMSLRALAGVWRMGTGIGPWSFLRGL
>CsMATE53
MADKDLDFSSHKLPTPSQVVEEMKELWSMALPITAMNCLVYIRAV
VSVLFLGWLGLASGLEPVCSQAYGNKNFDLLTLSLHHPDITSTAA
SYCIVSLPDL
LTNSFLQPLRVYLRSQGVTQPMMWCTFIAVVFHVPLNYVMVVVMK
LGVSGVALASVLTNVNMLVFLCVYVYVYGRWEWNWKWTAGIGGGG
VGPLLRLAVP

SCIGICLEWWWYEIVTVLAGYLPNPRLAVAATGILIQTTSLMYTV
PMALAGCVSARVGNELGAGKPYKAKLAAMVALGCAFLIGIIHVCW
TSIFRDKWAG
LFTKDEMLKALVASVMPIMGLCELGNCPQTTGCGILRGTARPAVG
ARINLGSFYFVGTPVAVGLAFWFSVGFSGLWLGLLSAQVACAISI
LYAVLVSTDW
EGEALKAMKLNELEMVEKCENGEENTRFLAKANGNTNC
>CsMATE54
MAENGVVHYPAEKGKMPIWVFFRDLRLLFRMDSLGSEILRIAFPA
ALALAADPVASLIDTAFIGRLGAVQIAAVGVSIAIFNQASKITIF
PLVSITTSLI
AEEDTNARLAKEMQKNNETVKVKAEDEILDSLEKGGSAAKTETKE
VKDEHTTVFVLTSASETTASKSQDVDCRDSYKIQAKAKKQQSTDL
VPNAPIEKRQ
LPSASTALIMGAVLGIFQTVFLISLAKPLLSTMGIKSNSPMLEPA
ERYLRLRSLGAPAVLLSLAMQGVFRGFKDTTTPLYATVAGDLANV
VLDPILIFFC
HMGVSGAAVAHVLSQYLILAILFCKLIKQVHLLPPSIKSMQLGVY
LLGRVIAATFCVTLATSLAARLGTTPMAAFQICLQVWMTSSLLSD
GLAVAGQAII
ACAFAEKDYQKAASAASRVLQMSIVLGLGLSLVVGLGMQYGSVVF
SNDKHVLYIISIGIPFVAATQPINSLAFVFDGVNYGVCDFVYSAY
SMVVVALLSI
ASLFFLSKSNGFVGIWLGLTIHMSLRALAGVLRMGSGTGPWRFLR
EI
>CsMATE55
MPHLVFFNDVRLLFKMDALGSEILHIADPVASLIDTVFIGRLAEE
DSIERLTKEVQNINEMVEVKAENVILDNLEKGGYYKRKETYSLSI
HSIGYGISSK
CGFLTPFLYLFSIWRVSGAAMAHVLSHPDVAHIWHIGHVCCFLTR
YHFIGFYLLVRMIAATFCVTLAASLASRLGPTPMAAFQICLQVWM
TPSLLADGLA
VAGQVLALLVIFSKSNPRIYEVKGIMSNMEGLRYSCYRLSLLVHL
LRSIIKRQPLLHLEYYSSTMAEDNVMHVPATQGKMPLLVFFRDLR
LLFKTDALGS
EILCIAFPATLALIADPVASLIDTTFIGHLGTVEIAAVGVSIAIL

NQALRITVSPLVSITTSFVAEEYAIGRLAKEKQKNKEIEKVKAED
VVPDSLEKGY
SATNSENIEAKSDNAILSDSNLSCETTATESPNVGCSVLNKTQIT
SKEQIIDLETDAVVMQVITPEKRHIPSASTSLIMGLVVGILQTIF
LISLAKPLLR
IMGVRSHSPMLDPAITYLRIRSLGFPAILLYLAMQGVFRGFKDTT
TPLYAIVAGDLANVVLDPILIFVCRMGVSGAAVAHVLSQCPETFC
ASCTFAVFLQ
GTIRLGRVIVATSCVTLATSLAARLGSTPMAAFQICLQVWMTSSL
LAYGLGGGGQAIIACALAEKDYQKAASTASRVLQMAFVLSLGVAL
VVGLGLQYGS
VVFTRDKHVLQIIAIGVPFVAATQPINYLAFAFDGLYYGVSDFAY
SAYSMVIIVILSIASLFLLWKSNGFAGIWLALTIFMSLRVLAGAL
RMGTGTGPWR
FLRSL
>CsMATE56
MSARSSVSEKHDDEASSELENILSNMNVSTWQRYQSATWIEMKLL
VYLAAPAVAVYMINYLMSMSTQIFCGHLGNLELAAASLGNTGIQV
FAYGLMLGMG
SAVETLCGQAYGASKFELLGIYLQRSAVLLTATGVLLTFAYIFSK
PLLILLGESTDISAAAALFVYGLIPQIFAYALNFPIQKFLQAQSI
VAPSAYISTV
TLVLHLIISYLVVYKIGLGLLGASLTLSLSWWIIVVAQFVYIVKS
ERCKHTWTGFSLTGAFSGLPAFFRLSAASAVMLCLETWYFQILVL
LAGLLENPEL
ALDSLSVW
>CsMATE57
MLFLGHLGDTELAAGSLAMAFANITGYSVLSGLALGMEPLLSHLR
LGVAGVAAASAASNLLVLVTVICYVWATGIHVATWSAPTRECLTG
WMPLIRLAAP
SCVSVCLEWWWYEIMIVLCGLLVDPKATVASMGVLIQTTSFIYVF
PSSLSFAVSTRVGNELGANHPDRARVSALVSIFLAGLMGFCAMSF
AIAVKDVWAH
MFTSDANIIKLTSTVLPILGLCELGNCPQTIGCGVVRGTARPSTA
ANVNLGAFYLVGMPVAIGLGFWFELGFSGLWLGMLAAQFCCAGLM
LCAVGSTKWD

DQARKAQALTCSGSEETRMPAGGDKEPLICITVT
>CsMATE58
MGMSCALETFCGQSYGAKQYHMLSIYMQRAMFVLSLVSVPLAFVW
ANTGIILKARGQDPAISEEAGRYAQYMIPSLFAYALLQCHVRFLQ
TRNIVFLMRF
SSSCAKTWTGFSKESLQNSPTFIRLAVPSAVMVCLETWSFEMLVL
LSGLLPNPQLETSVLSVSLNTAANCWMIPFGLGASIRSSTNCTFG
SVCGLCHGYY
GGVLVGVVLLLIRNIWGYAYSNEVEVVRYVAIMMPILATSNFFDG
IQCVLSGVVRGCGFQKFGAYINLGAYYLVGLPCAVLFAFVLHIRG
QNPAHLFDEV
PLVGDPLRIISSTERQNIEFTILQSLLK
>CsMATE59
MGMSCAMETFCGQSYGAKQYHMLGIHMQRAMFVLSLISIPLAFIW
ANTGIILKALGQDPAISEEAGRYAQYMIPSLFAYALLQCHVRFLK
TQNIVSPMMF
SSSCAKTWTGFSKESLQNIPTFIRLAVPSAVMVCLNTAANCWMIP
FGLSASISTWVSNKLGAGHPQTARSAMYVVFVMAITEGVLVGVVL
LLIRNIWGCA
YSNEVEVVRYVAIMMPILATSNFFDNIQCVLSGVVRGCGFQKFGA
YINLGAYYLVGLPCAVLFAFVLHIRGQTKKAEHRVHDSAIPVEVI
S
>CsMATE60
MEANDGLMSSAADALLEKEKNEGDMRCCSWSYKFLDMEEAKRQLQ
LASSNLANSWATVTGFSFMCSVPAEEIELLLKLCFEPILGLLHQD
PEISKAAALY
IKYLIPGLFAYGITYALVYWTSLGFIGAPLAASISLWISVLMLAS
YVLYAKKFKRTWEGFSWESSYHVFGNLKLAIPSAAMVCLEYWAFE
LLVLLAGIMP
NADVTTSLIAMCVNTEAVAYMFTYGLSASASTRVSNELGAGNPDK
AKHAMKVTLKLSIILAVAVVLALGFGHDIWAASFSNSSIIIEEFA
SVTPLLVISI
VVDSIQGILSGVARGCGWQHLAVYINLAMFYVIGMPIAMLLGIMV
GINLRTYLSSGQSAIANKVRCME
>CsMATE61
MAVSLMYGGGTSTRLLTRELSPSTAMGRSLSLQSSSQFRNSYTLV

DGKRLRYIGIVPRASLYTNYTQMWSPLPVMRQRRIPLLEINYKLS
SDSSVRTSDM
DESFSTEEEFVPSTSEDTFIDLNEISTSVASDKTKAELKGNATNP
THELVMLSLPAILGQAIDPFAQLMETAFIGRLGPVELASAGVSMS
IFNIVSKLFN
IPLLSVATSFVAEDISKNASSISTSVQGHEEINSARPLDAMARRE
QLSSVSTALTLAIGIGLFEAVALWLGSGVFLNLMGLSSVSFSHPK
VDIVPKISVY
SSCPASSMHAPARHFLSLRAIGAPAVVVSLALQGIFRGFKDTKTP
VICLCIGSAATILLLPVLMYYFKLGVTGAAISTVVSQYIVTCLML
WHLNKRAVLL
PPKLGSLQFGGYLKSGGFLLGRTLAVLITTTIGTSMATRQGPVAM
AAHQICLQVWLAVSLLTDALAASGQALISGSVSKGDYGSVKEVTY
SVLKIGFGTG
VLLTLLLGASFSSLATLFTKDAEVLAIVRTGVLFVSASQPINALA
FIFDGLHYGVSDFKYAACSMMVVGAISSMFLYCAPSVFGLPGVWA
GLTLFMGLRT
LAGVIRLLSKDGPWWFLHKDSTRLEFSAVA
>CsMATE62
MEGGVNENLLTEVRRAEEEGNLKDRVWTECKKMWIVAGPAIFSRF
STLGVSIISQAFIGHIGPTELAAYALVSIVLLRFANGIQLGMLAH
CNAMWPIICP
ILIALGQEESIAEVAGTISLWLIPGLDLWYNTILVLLAGSMKKAD
ISIDALSICLNINYWEMMISLGFMAAASVRVSNELGRGSSRAAKF
SIVQILLTAF
AIGFALFMFFLFFQGPVTYIFTESTEVAAAVDDLSPLLACSILLN
SIQPVLSGVAVGAGWQITVAYVNITGCYLIEIPIGVGVWVGMLLG
TLSQTTVLVI
ITYKTDWEKQVSIAEQRINKWSVDTDQESDADELNA
>CsMATE63
MGMSCAMETFCGQSYGAKQYHMLGIHMQRAMFVLSLVSMPLAFVW
ANTGIILKALGQDPAISEEAGRYAQHMIPSLFAYALLQCHVRFLQ
TQNIVFPMRV
ASGITTLLHVFLCWVLVFKSGLGCRGAALANSISYWINVLLLAFH
VKFSSSCAKTWTGFSTESLQNIPTFIRLAVPSAVMVCLNTAANCW
MIPFGLGASI

RSSTNCTFGGVCGLCHGYYGGVLVGVVLLLIRNIWGYAYSNEVEV
VRYVAIMMPILATSNFFDGIQCVLSGVITNHFQNPAHLFDEGPLV
KDPLRIISSS
VLPSYRHYTYQLE
>CsMATE64
MGMSCAMETFCGKSYGAKQYHMLGIHMQRAMFVLSLVSIPLAFIW
ANTGIILKALGEDPAISEEVGRYAQYMIPSLFAYALLQCLVRFLQ
TSISDDGKLR
NQNSATCLSLLDPSVQFSSSCAKTWTGFSKESLQNIPTFIRLAVP
SAVMVCLEMWSFEMLVLLSGLLPNPQLETSVLSVSLNTAANCWMI
PFGLSASIST
RVSNELGTGHPQTARSAVYVVFVMAITEGVLVGVVLLLIRNICGY
AYSNEVKKAEHRVHDSAIPVEVIS
>CsMATE65
MKIVSFISYHHLSCDQSIFHFNNHRSFTYTTVPPSKKHLQLVSLR
QPSCSFRLKSSSENNPSTKTETPHQENEKQVISSNRVKKPESEST
PVSFVFSSLS
FLRDGLKLDEVGWEIVSIALPAALALAADPLTSLIDTAFVGHLGS
VELAAVGVSASVFNLVSKLFNVPLLNVTTAFVAEEQAFITKGCDH
SIGSDQKGLQ
DKKVLPSVSTSLVLAATIGVAEAVALSVGSSFLMNTMGIPFDSPM
RVPAEQFLTLRAFGAPPIVIALAAQGTFRGFKDTKTPLYAVGAGN
LLNALLDPIL
IFLLGLGIGGAAISTVISEYLIAFILLWNLNDKVSLISPKFEATK
VFQYLKSGGFLIGRTVAVLVTMTVATSMVARDGPVPVAGYQICLE
VWLALSLLND
ALAIAGQLGITAGVSLTVFLLLGFGAITIFFTNDSKVLEIARSGT
LFVAGSQIMNALAFVLDGLYYGVSDFEYAAYSMVMIGLISSLFIL
AAAPLYGLAG
VWTGLYLFMTLRVVAGIWR
>CsMATE66
MEAAAAHTYTYCSLSTYAKTTFAAARRGRYHPHTYSVCSPRFINN
CSDHYHSLLFVPHLSKRRLVQCSSEGTVAISETSNDTPSVQTELI
RLSLPAIAGQ
AIEPLAQLMETAYIGRLGSLELASAGVSMSIFNIISKVFNIPLLS
VATSFVAEDISKHSTEGLSSAERMQLPSVSTALVLSIGIGILEAT

ALYVGSGMFL
SIMGISTASPMRISAHHFLQLRALGCPAVVLSLAIQGIFRGFKDT
ETPAVKDISGLLFILTSDNLLRTLGNTAAVFLFPTLMYVFHLGVT
GAAISSILSQ
YIVTLSMIWHLNKQTVLVPDLEKLQFGGYLKSGGFLLGRTLAAVM
TVTLSTSMAARQGPLSMAAHQIILQVWLSTSLLADAQSSSSQALI
ASSFAKGDYN
RVREITYISIKAGLFTGILLAIILSLSFGSLATLFTKDVEVLNIV
RSGLLFISASQPITALAYIFDGLHYGISDFMFAACSMMVVGALSS
AFLLWAPPLI
GLSGVWSGLTLFMALRTVAGLMRLSSKNGPWGFLQDIHGTEAAV
>CsMATE67
MLQQGNVRVGNELGAGHPKSAAFSVVIVNTVSFIISVIASIIVLL
LRDVISYAFTDGESVAKAVSDLCPLLALTLLLNGVQPVLSGVAVG
CGWQTFVAYV
NVGSYYVVGIPLGIVLGFYFNFGAKGIWLGMLGGTLMQTFILLWV
TFRTDWNREVVQAKKRLDNWDTKKQPLLVE
>CsMATE68
MFGGHLGEVYLLSASMATSFAGLRMGSALETFCGQAYGAKEYHMI
GVHMQRAMLVLMLICIPISILWSFNSDIFTFLGQDPEISVQSGAA
ISSGISYWVN
VLILGIYIKFSPTCEKTWTGYSMEGVKNLSTFLSVGIPSSLMLCL
EFWSYEFLVLMSGLLPNPKLETSMMSICLNTCSVFFRIPYGFGSA
EIHVQQSKQY
T
>CsMATE69
MCQLTSFPLSYKCNIDQSSYLINPVSKNLEPDMFAALIPNSTTTK
AKPSSLLSFHDLSDLPGPVIGHPVAQREKNSLVMSKFQAASRCLE
WWWYEIMILL
CGLFVNPRTTVASMGNLMQTTSLIYIFPSSLIFSVSTRVGNKLGA
NKPAKAKFAAIVGISCSCLLGLLALFFAVSVRNVWATMFTHDEDI
VALTSLVLPK
IGPCELGNCPQTTGCGVLRGTARPKIAANINLGCFYLVGMPVALG
LGFFMGIDFKGFGWVYWLH
>CsMATE70
MFPSTLLGTDQNWTLMKTISVTEYLNQIVIWFIIPAIRSSHADQN

NNALVLIVLLQYIPRLYLIFPFSSQIIKATGVIAKTAWAGAAYNL
VLYMLASHVL
GASWSLLSIERHAGCWKSAYRGDQDPVKCFPRYLDCRTFNDDDRR
TWANSKNVFQKCEPGNNEEFKYGIFENAVANHVVSSKCLEKFFYC
LWWGLQNLSS
VATLAFHVPVCWALIFKFNMGSDRAALAIGLSYWFNAVILGLYAM
YSSKCVETRVPFFMEVFSTIKEFFQFGIPSALMICLEWWAYEVII
FLAGVMKDPQ
LETSVLSIRHFNASTFLPALCFVTCIRVARGTGWQHLAAYVCLGS
YYLVGIPVALLLGFLVHLRAKGLWIGLLGGGLL
>CsMATE71
MVCLEMWSFEMLVLLSGLLSNPQLETSVLSVSLNTAANCWMIPFG
LGASIRSSTNCTFGGVCGLCHGYYGGVLVGVVLLLIRNIWGYAYS
NKVEVVRYVA
IMMPILATSNFFDGIQCVLSGVVRGCGFQKFGAYINLGAYYLVGL
PCAVLFAFVLHIRGQAKKAEHRVHDSAIPVEIIS
>CsMATE72
MSQFSGLLFLSLIWEVIEQHCLAICLSYWFNAIILGLYAMYSSKC
VETRVPFSMEVFSTIKEFFQIGIPSALMICLEWWAYEVIIFLAGV
MKDPQLETSV
LSISITVAFLHTFAPYSFSVAASVRISNELGAGNAKAVQRTVSVV
LVLGIIEAGISALVLFSLRQHSCDTFW
>CsMATE73
MFPSTLLGTDQNWTLMKTISVTEYLNQVLGASWYLLSIEMHAGCW
KSACRGDQDPVKCFPRFLDCRTFDDDDRRTWANNTTVFQKCEPGN
TEEFKYGIFE
NAVANHVVSSKFLEKFFYCLRWGLQNLRSHGEGQAKAGTEACNEH
FWRGKCPSSIATLDFHVPVCWALVFKFNMGSDRAALAIGLSYWFN
TVILGLYAMY
SSKSAETRVPFSMEVFSTIKEFFQIGIPSALMICLEWWAYEVIIF
LAGVMKDPQLETSVLSISVRISNELGDGNAKAVQRTVWVVLVLGI
IEAGIPALVL
FSLRYILGRAFISDNQMELQEEPDGSVLRLMLV
>CsMATE74
MIVSTASGYGSIIPDAPGQAKAGTEAYNEHFWRGKCLSSICWALV
FKFNMGSDRATLAIGLSYWFNDVILGLYAMYSSKCAETRVPFSME

VFSTIKEFFQ
IGIPSALMICREWWAYGVIIFLAGVMKDPQLETSVLSIRHFSAST
FLPALCFGTCIRVERETGWQRLAAYVCLGSYYLVGIQVALLLGFM
VHLRAKGLWI
GLLAGGLL
>CsMATE75
MRFSFSFAKTWTGFSKESLQNIPTFIRLAVPSAVVVCLEMWSFEM
LVLLSGLLPNPQLETSVLSVSLNTAANCWMIPFGLGASIRSSTNC
TFGGVCGLCH
GYYGGVVRGCGFQKFGAYINLGAYYLVGLPCAVLFAFVLYIRGQE
KKAEHRVHDSEIPVEVIS
>CsMATE76
MFPSTLLGTDQNWTLMKTISVTEYLNYEVAKAGTEACNEHFWRGK
CLSSICWALVFKFNMGSDGAALAIGLSYWFNAIILGLYAILEWWA
YEVIIFLAGV
MKDPQLETSVLSTSITVALLHTFAPYSFSVAASVRISNDLGAGNV
KAIQRTVWVVLVLGIIEAGISALILFSLRYVLGRAFELQEEPDGS
VLLLMFV
>CsMATE77
MVCLEMWSFEMLVLLSGLLPNPQLETSVLSVSLNTATNCWMIPFG
LGASIRSSTNCTFGGVFGLCHGYYGGVSVGVVLLLIRNIWGYAYS
NEVEVVRYVA
IMMPILATSNFFDGIQCVLSGVGAALKTADVETGSTVAIFGLGVI
GLAVAEGARLCGAKRIIGVDVNEDKHEVGKKFGLTNFVNSRNCGD
KPMSQLLGGM
RGMTRLLWKTSLLDPEEGIRFRDMSIPECQKLLPGAKSGGEPLPE
GLLWLLLTGKVVK
>CsMATE78
MGSDRAALAIGLSYWFNAVILGLYTMYSSKCVETRVQFSMEVFST
IKEFFQIGIPSALMTCWGNEGSPTGDFSALYKYYSRSPAYFCPYS
FSVASSVRIS
NELGARNAKAVQRTVWVVLVLGIIEACISALVLFSLRYFGMCIRV
ARGTRWQRLAAYVCLGSY
>CsMATE79
MKLNQNSALHCPEPQGIVTGNKRSNNSNEVEVVRYVAIMMPILAT
SNFFDGIQCVLSGVVRGCGFQKFGAYINLGAYYLVGLPYAVLFAF

VLHIRGQENQ
NGQGNQNDQGEQQNQDGHGNQLESFAWLERFSKQKPESFHSAPTP
IEAENWIVHLEKIFDALGCDELQKVRLAVYKLEGDAQRWWRGVKA
TRGEEFVETL
TWQGFKEVFYEQYFSNADREAYLREFHSITQNLDESITDYMARFI
RLAGFAGTIAGTAAQQAEKFKWGLKLNLRGPIISLKFPNVAEVAD
AAKDIEKERT
EYRAYKFQNDRKRTRDDQGSVQGRQWNGGQGSQHGQWRGRYQNTR
TQPAYGQNQYGGQKQNQNQQFQR
>CsMATE80
MLVLLSGLLPNPQLETSVLSVSLNTAANCWMIPFGFGASIRPSTN
CTFGSVCGLCHGYYGGVSVGVVLLLIRNTLGYAYSNEVEVVRYVA
IMMPILATSN
FFDGIQCVLSGVGAALKTADVETGSTVAIFGLGVIEGARLRRAKR
IIGVDVNEDKHEVGKKFGLTDFVNSRNCGDKHVSQVLGGMRGMTG
LFWKTSLLDP
EEGIRFRGMSIPECQKLLPGAKSGGEPRGSPLTSFNRKGNLLARM
MKMALFTIGTTS
>CsMATE81
MLVLLSGLLPNPQLETSVLSVSLNTVANYWMIPFGLDASIRSSTN
CTFGGVCGLCHGYYGGVSVGVVLLLIRNIWGYAYSNEVEVVRYVA
FMMPILATSN
FFDGTQCVLSGAALKTADVETGSTVAIFGLGVIGLAVAEGARLRG
AKRIIGVDVNVDKHEVGTWRYERKTGLLWKTSLLDPEEGIRFRGM
SISKCQKLLP
GAKSGGEPLPVGLL
>CsMATE82
MVEIVKFSGPATGLWLCGPLMSLIDTVVIGQSSSVELAALGPGTV
LCDNMSYLFMFLSVATSNFVATALAGKDKDEVQHQISILLFLGLA
CGVLMFFFTR
FLGARALTAFTGAKNVDIISAANTYVQIRSLAWPAVLIGSVAQSA
SLGMKDSWGPLKALSVASVVNGIGDVVLCTFLGYGIAGAAWATMA
SQVIAAYMMV
EALNKKGYNGYIFSVPSINDLKHIYSLAAPVFVTMISKVTFYSLL
VYFATSLGIQTVAAHQVMIQIFCMCAVWAEPLSQTAQSFMPELIH
GANQNLSKAR

MLLKSLLIIGALSGTILGSIGTAVPLLFPQAFSPDAEVIMEMHKV
LIPFFIALCVTPCTSSLEGTLLAGRDLKYISLSMGVIVAFGTLLL
MIFSGRGTGL
SGCWWALAAFQWCRFSSALRRLNLSSGILYSDTLMPRNILTHEIS
>CsMATE83
MEEPLLPMANNRQKSQGAASVVVEVETLSSALPKRWEVIVKEVKR
MSYIAMPMVVTTLSENMVRIVISMMMIGHLGELSLSGAAIATSLT
NVTGFSLLVP
SLSSSISSYMNINAQVIK
>CsMATE84
MIDDIKRCLDVGNELGAGKPYKAKLAAMVALGCAFLIGIIHVCWT
SIFRDKWAGLFTKDEMLKALVASVMPIMGLCELGKLPPNHRLWHP
TWHRETSSGS
LISSSGLRDIYLIRRVSEHGLEGEALKAMKLNELEMVEKCENGER
EY
>CsMATE85
MNLKSLVTTTFPNPKFLNVFSKQSFAPPPKHLLVSSTTRRFTTCL
RSSDDNVVFAANDSLWSQIVEIVSFSGPATGLWACGPLMSLIDTV
VIAQASSLQL
AALGPGTVLCDDLGYVFMFLSIATSNLVATALAGKGKDEVQHQIS
ILLFVGLTCGVLMLLFTRFFGAWALTAFTGAQNVDIIYSANKYVQ
IRGLAWPAVL
IGWVAQSASVGLKDSWGPLKALIVASAINGIGDVVLCTFLGYGIA
GAAWATMASQVVAAYMMVEALNNKGYNGFALSVPSFGELQHIYML
AAPVFVLMMS
KVAFYALLVYFATSMGVQAVAAHQVMIQMYFMCAVWAEPLAQTAQ
SFMPELLYGAKRSLLKARVLLKSLVIVGVLSGIILGSIGTVIPWL
FPFAFSPDKE
ITKEMHKVLVPYFITLCVTPSTSSLEGTLLVSLFYRVSSVF
>CsMATE86
MKTISVTKYLNYGAGKFSKSKASGYGSIIPVEPGAESHLLTKNLG
DKVGNCVEEYIEAMEKVKLKQGLKLAMRISGDGNAYLQKLKIVLY
VNALCSDGAA
LAIGLSYWFNAVILGLYAMYSSKCAQTRIPFSMEDSSTIKEFFQI
GIPSAIMICWGNEGSPTGDFSALYKYYSRSSAYFFPILFFGCSKE
LQEEPDGSVL

RLMFV
>CsMATE87
MSKTTTYLANLLQTCIDKKSHISGKLIHAYILRHGLYADTFLSNR
LIELYNKCNKINTASHLFDQMSLKNIYSCNAMLSAYCKSNKLEDA
HVLFDEMPER
NVVSWNTLISALVRNGMAEKALGVYCRMKWEGFVPTHFTLASVVS
ACGVMVDVECGRGEHCVAIKIGLDENVYVGNALLSMYAKCGCVGD
AVKAFEDLVE
PNEVSFTAMMGVLAETDRVEEALKMFRGMFRIGISVDSISLSCVL
GVFSRSGIGNTKGLLGNAHGRLIHGLIIRLGFEKDLHLGNSLLDM
YAKTRDMESA
EAVFTYLPELSIVSWNVMIGGYGQDYQIDKALKYMRMMKNYGFEA
DEVTHVNMLAACIKSGNVETARKIFSNMACPSLASWNAMLSGYFQ
NEKLKEAIKL
FREMQFRHVQADRTTLAIILNSCAALGLFEWGKSVHCTSIKGLNH
TDIYVGSGLIGLYSKCNQADVAKSIFDRMQDRDVVCWNSMIAGLS
LNSLDKEAFN
LFKQMIGKGLYPTQFSYATILSSCAKSSSLPQGRQIHAQIVKDGG
VNDVYVGSALIDMYSKCGEVNEARIFFDTMPFRNTITWNEMIHGY
AQNGCGNEAV
DLFDEMIQSGESLDGITFLAVLTACSHSGLVDHGVAIFNSMQSEH
GVEPLSDHYTCMIDTLGRAGRFHEIEVLVDKMPCNDDPIVWEVLL
SSCRVHDNVS
LARRAADALFSLDPKNSAPYVLLANMYSSLGRWDEVKDVRDMMSK
KSIVKNPGYSWLEHKSEKQHCMVDHYRYMEMHDFEAGFAYAKLCT
AVLKIEVSWC
SSYSNLTAMQGGFRGFKDTQTPPYATCFLLLMKVIALTFCVTLAA
SMAVNQGPIPMASFQVCLQVWLAMSIIADGLAVAGQVRNLELFRS
EY
>CsMATE88
MKLSVHKKLSIAAFLHSDQDGSSALGPVEFSSAGVSISIFNIVSK
LSNIPLLSVATSFVPKTFQKMHVQSLLQASSMHAPAQRFLSLRAI
GAPAIVVSLA
LKAFFGDSGHKNSYSKLNIERHVAIFITIAMVRALYLVTCLMIWN
LNMRAILLPPKLGSLKFAGYVKSVLITTTIGTSMATRQGPLEMAA
NQICIQVWLA

VSLMTDALATSGQLTLFVLCCLNGDPITGDGFW
>CsMATE89
MQAVTNGKCNGGWTQNKTGVKAYVAASVGISNELGAGNAKAVQRT
VWVVLVLGIIEAGLSALVLFSVRYVLGRAFELQEEPGGSVLRLMF
V
>CsMATE90
MDYISCAKFLIVREIVQQDKLAGWGYSAGGFLVASAINCSPHLFR
AAVLEVPFLDPRNTLLNPVIPLTTYDYEEFGYPVDVEDFDAIQRY
SPYDNIQKGV
LYPAVLVTTFFKTSLEWWVYEVIIFLVGVMKDPQLETSVLSIRWF
CQILINCRNNKKLLGRVRAFDRHCNMVMENVREMWTELPKAGKGK
KKSLPINKER
FISKMFLRGDSVIIVLRNPK
>CsMATE91
MGNPNKDLDMSHLAASLPTAAAGPGTVLCDNMSYVFMFLSIATSN
FVATALAGQDKNEVQHQISILLFVRLTCGVFMFFFTRLWGEWALT
AFTGAKNVEI
ISSANTYVQSLCKSCNGQRVVRGPKSVKLDIMPVIALGFSTPFSS
HPFLDCA
